# MBD3 Knockdown Promotes the Differentiation of Bovine iPSCs into Primordial Germ Cell-like Cells Associated with Altered Bivalent Modification of TGF-β-Related Genes

**DOI:** 10.3390/cells15171577

**Published:** 2026-08-30

**Authors:** Wen Yuan, Jing Wang, Weiqi Li, Yang Luo, Yiting Liu, Shun Long, Yiyang Wang, Fang Liu, Xiaoxiong Geng, Yongli Yue, Xueling Li

**Affiliations:** 1 State Key Laboratory of Reproductive Regulation and Breeding of Grassland Livestock, College of Life Science, Inner Mongolia University, Hohhot 010021, China; 20250069@imnu.edu.cn (W.Y.); nnlrl@mail.imu.edu.cn (J.W.); shun.long@guidonpharmaceutics.com (S.L.); 32308246@mail.imu.edu.cn (Y.W.); liufang@mail.imu.edu.cn (F.L.); 32208036@mail.imu.edu.cn (X.G.);; 2 College of Life Science and Technology, Inner Mongolia Normal University, Hohhot 010022, China

**Keywords:** MBD3, PGCLCs, bivalent

## Abstract

**Highlights:**

**What are the main findings?**

- *MBD3* knockdown during bovine iPSC reprogramming significantly enhances the differentiation efficiency of primordial germ cell-like cells (PGCLCs) under a 2D monolayer culture system.
- *MBD3*-knockdown iPSCs exhibit elevated bivalent histone modifications (H3K4me3/H3K27me3) at TGF-β-related gene loci and upregulated DPPA4/DNMT3L expression, which primes enhanced NODAL signaling to drive PGCLC formation.

**What are the implications of the main findings?**

- MBD3 functions as an epigenetic gatekeeper that restricts germline competence during reprogramming, and its depletion predisposes iPSCs toward a PGCLC fate.
- These findings provide a mechanistic basis for optimizing bovine iPSC-derived PGCLC protocols through targeted epigenetic modulation, with potential applications in livestock breeding and germline conservation.

**Abstract:**

Methyl-CpG binding domain protein 3 (MBD3) is a member of the nucleosome remodeling and deacetylase (NuRD) corepressor protein complex, which plays a pivotal role in embryonic development, pluripotent stem cell (PSC) differentiation, and induced pluripotent stem cell (iPSC) reprogramming. However, whether MBD3 is also involved in iPSC differentiation into primordial germ cell-like cells (PGCLCs) is unclear. In this study, bovine iPSCs generated by *MBD3* knockdown (*MBD3* KD) during reprogramming were subjected to PGCLC differentiation using a monolayer cell culture method. Our results revealed that *MBD3* KD enhanced the ability of bovine iPSCs to differentiate into PGCLCs. *MBD3*-KD bovine iPSC-derived PGCLCs exhibited the characteristics of in vivo primordial germ cells (PGCs) in a migratory state. *MBD3* KD increased NODAL signal transduction during the induction of PGCLCs and was associated with increased expression of *PRDM1* and *SOX15*. Moreover, *MBD3* KD increased the bivalent modification of transforming growth factor beta (TGF-β)-related genes concomitant with the upregulation of DPPA4 and DNMT3L expression in bovine iPSCs, and this epigenetic change was associated with enhanced NODAL signaling. Our results indicate that *MBD3* KD during reprogramming favors the differentiation of bovine iPSCs into PGCLCs, further highlighting the impact of MBD3 on histone modifications during germ cell development.

## 1. Introduction

MBD3, a member of the methyl-CpG binding domain (MBD) protein family [1], lacks a transcriptional repression domain and exhibits up to 70% sequence homology with its paralog MBD2, with which it competes as an alternative core subunit of the nucleosome remodeling and deacetylase (NuRD) complex [2,3]. As a core subunit of NuRD, MBD3 connects histone deacetylation, nucleosome remodeling, and selective recognition of methylated DNA into an integrated epigenetic machinery [4]. MBD3 plays important roles in the maintenance of imprinted genes during early embryonic development [5], the maintenance of ESC pluripotency and self-renewal [6], the differentiation of pluripotent stem cells (PSCs) [7], and the generation of induced pluripotent stem cells (iPSCs) [8,9,10,11]. Notably, the role of MBD3/NuRD in reprogramming is context-dependent: it can function as either a positive or negative regulator depending on the specific reprogramming system used [12,13]. Previously, we reported that the knockdown of MBD3 promotes the pluripotency and in vitro differentiation ability of bovine iPSCs by reducing DNA damage via HIF1A during reprogramming, suggesting that MBD3 depletion may enhance the developmental competence of bovine iPSCs.

A key mechanism by which MBD3/NuRD regulates developmental competence involves the modulation of bivalent chromatin modifications. In ESCs, a unique “bivalent” histone modification pattern—the simultaneous presence of H3K4me3 and H3K27me3 marks near the promoters of developmentally regulated genes—silences developmental genes while keeping them poised for activation upon lineage commitment [14]. This bivalent state is established through the recruitment of COMPASS family members (e.g., SET1/MLL) and PRC2 complexes to unmethylated CpG islands near developmental gene promoters [15,16]. MBD3 and NuRD have been shown to directly bind to bivalent promoters and mediate H3K27 deacetylation, thereby recruiting PRC2 to establish or maintain H3K27me3 marks [8,17]. This cooperative interplay between MBD3/NuRD and polycomb proteins is critical for maintaining the epigenetic plasticity required for germline specification.

The formation of primordial germ cells (PGCs) represents the first step in germline specification and serves as an essential benchmark for assessing the developmental competence of PSCs [18]. However, the signaling pathways and transcriptional hierarchies governing PGC specification differ markedly between species. In mice, PGCs are initiated by WNT-mediated BMP4 signaling, which activates PRDM1 (BLIMP1) and PRDM14, both acting with TFAP2C to induce PGC fate [19,20,21]. In humans, BMP signaling upregulates PRDM1 and TFAP2C, while SOX17 cooperates with PRDM1 to establish the human PGC gene expression program [18,19]. While the development of bovine PGCs appears somewhat similar to that of humans, the precise signaling pathways, key transcriptional regulators, and regulatory networks remain largely uncharacterized. Consequently, whether bovine iPSCs can efficiently differentiate into primordial germ cell-like cells (PGCLCs) and how *MBD3*-mediated epigenetic mechanisms influence this process are unresolved questions.

In this study, bovine iPSCs generated by MBD3 knockdown (MBD3 KD) during reprogramming underwent PGCLC differentiation. We first optimized the differentiation protocol to increase the efficiency of bovine iPSC-to-PGCLC conversion. The derived bovine PGCLCs were then characterized using quantitative real-time PCR (qRT–PCR), bulk and single-cell RNA sequencing (scRNA–seq), and immunofluorescence staining. Furthermore, the mechanism by which MBD3 knockdown affects PGC derivation was investigated through a chromatin immunoprecipitation sequencing (ChIP-Seq) analysis of bivalent modifications and inhibition of relevant signaling pathways. Collectively, our results indicate that MBD3 KD enhances NODAL signaling during bovine PGCLC induction, promoting PRDM1 and SOX15 expression by increasing bivalent chromatin marks on TGF-β-related genes via the upregulation of DPPA4 and DNMT3L in iPSCs. Our study provides a new perspective on obtaining PGCLCs from iPSCs.

## 2. Materials and Methods

### 2.1. Induction of PGCLCs from Bovine iPSCs (2D)

Bovine iPSCs were generated and cultured using the LCDM system according to previously published protocols [1]. The LCDM contained equal amounts of DMEM/F12 (11330-033, Gibco, New York, NY, USA) and neurobasal medium (21103-049, Gibco, New York, NY, USA) supplemented with 0.5% N2 supplement (17502-048, Gibco, New York, NY, USA), 1% B27 supplement (17504-044, Gibco, New York, NY, USA), 1% L-glutamine (Sigma-Aldrich, St. Louis, MO, USA), 1% MEM nonessential amino acid solution (NEAA; M7145, Sigma-Aldrich, St. Louis, MO, USA), 0.1 mM β-mercaptoethanol (Sigma-Aldrich, St. Louis, MO, USA), 1% penicillin–streptomycin (PS; 15140122, Gibco, New York, NY, USA), 5% knockout serum replacement (KSR; 10828028, Gibco, New York, NY, USA), 10 ng/mL recombinant human leukemia inhibitory factor (LIF; 300-05, Peprotech, Cranbury, NJ, USA), 1 µM CHIR99021 (HY-10182, MCE, Monmouth Junction, NJ, USA), 2 µM (S)-(+)-dimethindene maleate (1425, R&D Systems, Minneapolis, MN, USA), and 2 µM minocycline hydrochloride (HY-17412, MCE, Monmouth Junction, NJ, USA).

#### 2.1.1. Bovine Fetal Fibroblast (BFF) Isolation and Culture

BFFs were isolated from ear and tail biopsies of bovine fetuses obtained at slaughterhouses. Cells were maintained in DMEM (Gibco) containing 10% fetal bovine serum (FBS; 50325, Bovogen, Melbourne, Victoria, Australia) and 1% penicillin–streptomycin (Gibco, 15140122, Gibco, New York, NY, USA) at 38.5 °C under CO_2_. The medium was refreshed daily, and cells were subcultured upon reaching ~90% confluence.

#### 2.1.2. Animal Experiments

All animal procedures were conducted in accordance with the guidelines set forth by the Institute Animal Care and Use Committee and were approved by the Animal Care and Use Committee of Inner Mongolia University (approval code: IMU-MOUSE-2021-047). The mice of the CD1 (ICR) strain were obtained from Beijing Vital River Laboratory Animal Technology (Beijing, China). All mice were maintained in specific pathogen-free (SPF) conditions, with a 12 h dark/12 h light cycle.

#### 2.1.3. Reprogramming

BFFs were electroporated with a cocktail of four PiggyBac transposon plasmids—CAG-bovine *OCT3/4*, CAG-bovine *SOX2*, CAG-bovine *KLF4*, and CAG-bovine *c-MYC*—using ~10^6^ cells per reaction. Post-electroporation, ~5000 cells were seeded per well of a 12-well plate pre-coated with feeder cells in DMEM/10% FBS. After 24 h, the medium was switched to LCDM for reprogramming. Emergent iPSC colonies became visible between days 12 and 23 and were subsequently picked and expanded using TrypLE Select.

#### 2.1.4. PGCLC Induction

Bovine iPSCs were differentiated into iMeLCs and PGCLCs using a modified protocol from a previously published method [2]. For iMELC induction, the iPSCs were dissociated into single cells with TrypLE Select (Gibco), and 1 × 10^5^ cells per well were plated onto Matrigel (354234, Corning Incorporated, Tewksbury, MA, USA) -coated 12-well plates in Glasgow minimum essential medium (GMEM; 11710-035, Gibco, Grand Island, NY, USA) supplemented with 15% KSR (KSR; 10828028, Gibco, New York, NY, USA), 1% NEAA (M7145, Sigma-Aldrich, St. Louis, MO, USA), 2 mM L-glutamine (Sigma-Aldrich, St. Louis, MO, USA), 1 mM sodium pyruvate (11360-070, Gibco, Grand Island, NY, USA), 0.1 mM β-mercaptoethanol (Sigma-Aldrich, St. Louis, MO, USA), 50 ng/mL Activin A (338-AC-010, R&D Systems, Inc., Minneapolis, MN, USA), 1 µM CHIR99021 (HY-10182, MCE, Monmouth Junction, NJ, USA), and 10 µM ROCK inhibitor (Y-27632; HY-10583, MCE, Monmouth Junction, NJ, USA). After 2 days of culturing, the cell culture medium was replaced with GMEM supplemented with 15% KSR, 0.1 mM NEAA, 2 mM L-glutamine, 1 mM sodium pyruvate, 0.1 mM β-mercaptoethanol, 200 ng/mL bone morphogenetic protein 4 (BMP4; HY-P70023, MCE, Monmouth Junction, NJ, USA), 100 ng/mL stem cell factor (SCF; 300-07, PeproTech, Cranbury, NJ, USA), 20 ng/mL LIF (LIF; 300-05, Peprotech, Cranbury, NJ, USA), 50 ng/mL epidermal growth factor (EGF; 236-EG, R&D Systems, Minneapolis, MN, USA), and 10 µM ROCK inhibitor (Y-27632; HY-10583, MCE, Monmouth Junction, NJ, USA) for PGCLC induction. The medium was replaced every day, and the culture was continued for 6–8 days.

### 2.2. Quantitative Real-Time PCR (qRT–PCR) Analysis

Total RNA was extracted using the Eastep™ Super Total RNA Extraction Kit (LS1040, Promega, Madison, WI, USA) following the manufacturer’s instructions. A GoScript^TM^ Reverse Transcription System (A5000, Promega, Madison, WI, USA) was used for the synthesis of complementary DNA. Reactions for reverse transcription–polymerase chain reaction were conducted on a 7500 Real-Time PCR System (4351104, ABI Biosystems, Foster City, CA, USA) using GoTaq^®^ qPCR Master Mix (A6001, Promega, Madison, WI, USA). Gene expression was calculated using the 2^−ΔCT^ method and normalized to that of the housekeeping gene *GAPDH*, with the data presented as means and standard deviations. The sequences of the primers utilized are presented in the Supplementary Materials, Table S1.

### 2.3. Immunofluorescence Staining

The cells were washed once in DPBS, fixed in 4% paraformaldehyde (PFA; P1110, Solarbio, Beijing, China) for 15 min, permeabilized for 30 min with 1% Triton X-100 (IR9069, Solarbio, Beijing, China), and blocked for one hour at room temperature with 5% BSA. The samples were subsequently incubated with primary antibodies at 4 °C overnight. Following three DPBS washes, the samples were incubated for 1 h at room temperature with secondary antibodies. Following two washes, the samples were incubated for 3 min at room temperature with DAPI (0.5 mg/mL; C1006, Beyotime, Shanghai, China). Following two washes in DPBS, the samples were loaded onto slides with antifade solution (S2100, Solarbio, Beijing, China). Fluorescent signals were visualized using Nikon confocal laser-scanning microscopes (Nikon, A1, Tokyo, Japan). The antibodies utilized in this study are listed in the Supplementary Materials, Tables S2 and S3.

### 2.4. Western Blot

The cells were harvested and lysed with lysis buffer (89900, Thermo Fisher Scientific, Waltham, MA, USA) supplemented with phenylmethylsulfonyl fluoride (PMSF; ST507, Beyotime, Shanghai, China) on ice for 30 min. The supernatant was subsequently collected following centrifugation at 13,200 rpm for 5 min. The protein concentration was determined using the BCA colorimetric method. The samples were boiled for approximately 10 min, and the proteins were subsequently subjected to SDS–PAGE on 10% Bis-Tris gels (345-0111, Bio-Rad, Hercules, CA, USA) and transferred to NC membranes. The membranes were blocked for 90 min in 5% skim milk, after which they were incubated with primary antibodies overnight at 4 °C. The samples were subsequently incubated with secondary antibodies at room temperature for 1 hr. The target protein bands were then visualized by enhanced chemiluminescence (32106, Thermo Fisher Scientific, Waltham, MA, USA) and detected by an imaging analysis system (Bio-Rad, Hercules, CA, USA). The antibodies utilized in this study are listed in Tables S4 and S5 in the Supplementary Materials.

### 2.5. RNA Isolation

Total RNA was extracted from cells using a spin column-based RNA purification kit (LS1040, Promega, Madison, WI, USA) according to the manufacturer’s instructions. Briefly, cells were lysed in RNA lysis buffer and homogenized thoroughly. After adding the dilution buffer, the lysate was centrifuged at 12,000–14,000× *g* for 5 min at room temperature. The supernatant was carefully transferred to a new RNase-free tube, and 0.5 volume of absolute ethanol was added and mixed thoroughly. The mixture was then loaded onto a purification column and centrifuged at 12,000–14,000× *g* for 1 min. After washing with RNA wash buffer, on-column DNase I digestion was performed by adding 50 μL of freshly prepared DNase I incubation buffer to the column for 15 min to eliminate genomic DNA contamination. The column was then washed twice with RNA wash buffer and centrifuged at 12,000–14,000× *g* for 45 s each time. Finally, total RNA was eluted with 30 μL of RNase-free water by centrifugation at 12,000–14,000× *g* for 1 min. The purified RNA was stored at −70 °C until further use. RNA quantity and quality were assessed prior to library construction.

### 2.6. Bulk RNA Sequencing Analysis

Bulk RNA-seq experiments were conducted on iPSC-NC, iPSC-siMBD3, iMeLC-NC, iMeLC-siMBD3, PGCLC-NC-d3, PGCLC-NC-d8, PGCLC-siMBD3-d3 and PGCLC-siMBD3-d8 cells. A total of 1 µg of RNA was utilized as the input material for the RNA sample preparations. Sequencing libraries were generated using the NEBNext Ultra^TM^ RNA Library Prep Kit for Illumina (NEB, Ipswich, MA, USA), and index codes were added to attribute sequences to each sample. Each treatment was conducted in triplicate. The PCR products were purified, and the quality of the library was assessed using an Agilent Bioanalyzer 2100 system (Agilent, Palo Alto, CA, USA). The resulting libraries were sequenced on an Illumina NovaSeq platform (Illumina, San Diego, CA, USA), and 150 bp paired-end reads were generated.

For RNA-seq data analysis, the clean reads were mapped to the bovine reference genome using HISAT2 v2.2.1 to generate BAM files. Gene expression quantification was then performed on the aligned reads using FeatureCounts v2.0.1 [3]. The read counts of each gene were calculated, and the expression of each gene was standardized with TPM. DEGs were computed using the edgeR package in R (version 4.0.2) [4]. An adjusted *p* < 0.05 and |Log2 (fold change)| ≥ 1.5 were considered to indicate a significant enrichment of DEGs. Pearson correlation analyses, heatmaps, PCA, and hierarchical clustering were performed in R (v4.0.4). Enrichment analyses of the DEGs by GO and gene set enrichment analysis (GSEA) were implemented using the ClusterProfiler R package, which corrects for gene length bias [5]. A corrected *p* < 0.05 after calibration was considered to indicate a significant enrichment of DEGs.

### 2.7. Single-Cell RNA Sequencing (scRNA-Seq) Analysis

The concentration of single-cell suspensions was adjusted such that the proportion of viable cells exceeded 80% to 700–1200 cells/μL. The cells of each sample were then processed using the MobiCube^®^ High-Throughput Single-Cell 3′ RNA-Seq Kit (v2.1) according to the manufacturer’s instructions (Novogene Bioinformatics Technology Co., Ltd.; Tianjin, China). The cells were loaded onto the Chip A Single-Cell Kit (S050100201, MobiDrop, Jiaxing, China) to generate droplets with MobiNova-100 (A1A40001, MobiDrop, Jiaxing, China) microfluidic chips. Each cell was involved in a droplet that contained a gel bead linked with up to millions of oligos (cell unique barcodes). After encapsulation, the droplets were light cut by MobiNovaSP-100 (A2A40001, MobiDrop, Jiaxing, China) while the oligos diffused into the reaction mixture. The mRNAs were captured by gel beads containing oligo(dT) in droplets. Following reverse transcription, cDNAs with barcodes were amplified, and a library was constructed using the High Throughput Single-Cell 3′ RNA-Seq Kit v2.0 (S050200201, MobiDrop, Jiaxing, China) and the 3′ Single Index Kit (S050300201, MobiDrop, Jiaxing, China). Libraries were sequenced on an Illumina NovaSeq sequencer with 150 bp paired-end reads (Novogene Bioinformatics Technology Co., Ltd.; Tianjin, China).

For single-cell RNA-seq data analysis, single-cell suspensions were prepared from pooled multiple independent cell line samples for each condition (PGCLC-NC and PGCLC-siMBD3), without biological replicates. This pooling strategy was employed to minimize inter-sample variability and ensure a comprehensive representation of the cellular landscape for each condition. The raw data were analyzed with MobiVision (v3.2) using default parameters. The pipeline (https://www.mobidrop.com/bioinformatics-analysis-software/mobivision-news/software-download) (accessed on 15 June 2024) included the processing of raw data to align reads to the reference genome and generate gene–cell matrices. The genome and GTF annotation files of bosTau9 were downloaded from the Ensembl website (https://www.ensembl.org/) (accessed on 15 September 2024). The gene–cell matrices generated by MobiVision served as input data for further analyses with Seurat (v5.1.0). To remove dead and poor-quality cells, the following filtering thresholds were applied: genes expressed in fewer than three cells were excluded (min.cells = 3), and cells with fewer than 200 detected genes were removed (min.features = 200). Additionally, cells with mitochondrial gene content exceeding 10% (percent.mt > 10) or with total detected genes fewer than 200 or more than 6000 (nFeature_RNA > 200 and nFeature_RNA < 6000) were excluded. After filtering, a total of 23,257 cells (14,054 from iPSC-NC and 9203 from iPSC-siMBD3) were retained for downstream analysis. The scaled data were first normalized using sctransform (v0.4.1). For PCA, the scaled data were reduced to 50 approximate principal components (PCs) depending on the highly variable genes (set npcs = 50). Afterward, clusters were identified using the Seurat function “FindClusters” with appropriately selected PCs and “resolution = 0.2”. The cluster-enriched genes were identified using the Seurat function “FindAllMarkers” and tested with “wilcox”. The cluster-enriched genes were detected using the parameters “min.pct = 0.25” and “logfc.threshold = 0.25”, indicating that the minimum cell percentage for enriched genes was greater than 0.25 and that the log2-fold change in average expression was greater than 0.25. The single-cell data structures and trajectories were separately visualized and explored using UMAP.

### 2.8. Integration of scRNA-Seq and Bulk RNA-Seq Data

To enable a comparative analysis between single-cell RNA-seq and conventional bulk RNA-seq data, we first isolated the PGCLC subpopulation from the scRNA-seq dataset and constructed an independent Seurat object. Subsequently, using biological samples as the unit of aggregation, we employed the PseudobulkExpression function in Seurat to sum the raw counts of PGCLCs, generating pseudobulk expression matrices with a data structure comparable to that of bulk RNA-seq. The pseudobulk expression matrices were then matched with conventional bulk RNA-seq expression matrices by gene name, retaining only genes commonly detected in both datasets. Finally, the merged expression matrix was subjected to consistent normalization and necessary batch correction procedures, followed by correlation analysis, clustering analysis, and differential expression pattern comparison under a unified expression scale.

### 2.9. Reduced Representation Bisulfite Sequencing (RRBS) Analysis

#### 2.9.1. Library Preparation and Sequencing

A total of 5.2 micrograms of genomic DNA spiked with 26 ng of lambda was digested using the methylation-insensitive restriction enzyme MspI. DNA was fragmented through sonication to 200–300 bp with Covaris S220 (Covaris, Woburn, MA, USA), followed by end repair and adenylation. The cytosine-methylated barcodes were ligated to sonicated DNA according to the manufacturer’s instructions. These DNA fragments were subsequently treated twice with bisulfite using an EZ DNA Methylation-Gold^TM^ Kit (D5005, Zymo Research, Irvine, CA, USA), after which the resulting single-strand DNA fragments were amplified via PCR using KAPA HiFi HotStart Uracil + ReadyMix (2×) (Roche Sequencing Solutions, Indianapolis, IN, USA). Library quality was subsequently assessed on an Agilent 5400 system (Agilent, Santa Clara, CA, USA) and quantified using QPCR (1.5 nM). Pair-end sequencing of the samples was performed on an Illumina platform (Illumina, San Diego, CA, USA).

#### 2.9.2. Bioinformatics Analysis

Data Quality Control: Short reads (raw data) in FASTQ format were obtained, which contain sequence information and corresponding sequencing quality information. Ensuring reliable bioinformatics analysis requires overcoming sequence artifacts, such as reads containing adapter contamination, low-quality nucleotides, and unrecognizable nucleotides (N). To achieve meaningful downstream analysis, a crucial step involves quality control measures. In this study, we used Fastp (0.19.7) to perform basic statistical analysis on the quality of the raw reads. The data processing steps implemented were as follows: (1) Paired reads were discarded if either one of the reads contained adapter contamination. (2) Paired reads were discarded if more than 10% of the bases were uncertain in either one of the reads. (3) Paired reads were discarded if the proportion of low-quality bases (Phred quality < 5) exceeded 50% in either one of the reads. By applying these rigorous quality control measures, we aimed to ensure the reliability of the subsequent analyses and improve the accuracy of our findings.

Mapping: Using bwa-meth (0.2.2) software, the data treated with bisulfite were aligned to the reference genome. First, the reference genome was converted (C => T, G => A) and then indexed using bwa (0.7.17). Next, the sequenced reads were subjected to the same conversion and aligned to the converted reference genome. By comparing the best alignment sequences generated from the original forward and reverse strands with the sequences from the normal genome, the methylation status of cytosines can be inferred.

Extracting methylation levels: MethylDackel (https://github.com/dpryan79/MethylDackel, version 0.5.2) (accessed on 15 June 2024) was used to extract the methylation levels of all cytosine positions. All cytosines were classified into one of three sequence contexts: CpG, CHG, and CHH (H = A, C, T, N). If N was encountered in the reference sequence, it was assigned to either CHG or CHH. If a cytosine was located close to the chromosome end, making it impossible to infer the site, it was classified as CHH. The extracted CHG and CHH positions were subsequently combined to form the sample’s mCH level file.

### 2.10. Chromatin Immunoprecipitation Sequencing (ChIP-Seq) Analysis

ChIP experiments were conducted using anti-MBD3 (Cell Signaling Technology, Danvers, MA, USA), anti-H3K4me3 (Cell Signaling Technology, Danvers, MA, USA), and anti-H3K27me3 (Cell Signaling Technology, Danvers, MA, USA) antibodies and a SimpleChIP^®^ Enzymatic Chromatin IP Kit (Magnetic Beads) (9003, Cell Signaling Technology, Danvers, MA, USA). Following cross-linking and fixation of the cells with 16% formaldehyde (12606, Cell Signaling Technology, Danvers, MA, USA), the purified nuclei were subjected to sonication, resulting in the generation of chromatin fragments with a size range of 150 to 900 base pairs. The chromatin fragments were subsequently incubated overnight with anti-MBD3, anti-H3K4me3, and anti-H3K27me3 antibodies. The antibody-bound DNA fragments were then pulled down using Protein G magnetic beads and subsequently purified for library construction. Following quality control of the DNA sample, the purified DNA was subjected to ChIP-seq library preparation using the NEBNext^®^ Ultra™ II DNA Library Prep Kit (E7645L, NEB, Ipswich, MA, USA). The qualified libraries were pooled and sequenced on an Illumina platform with a PE150 strategy at Novogene Bioinformatics Technology Co., Ltd. (Beijing, China), according to the effective library concentration and data amount needed.

For ChIP-Seq data analysis, Illumina reads were first mapped to the UCSC bosTau9 reference using bwa-mem (version 0.7.17) with default parameters [6]. Next, we used macs2 (v2.2.7.1, -nomodel-broad-broad-cutoff 0.1-shift 0-gsize 2.7e9-keep-dup auto) to call the peaks [7]. MBD3 signals were normalized using the MA norm (version: 1.1.4) method for the quantitative comparison of ChIP-seq data [6], and the significantly different peaks were defined as those whose log10(*p* value) was <0 and whose M value was >1. The differential peaks were subsequently annotated to UCSC bosTau9 using the R package ChIP seeker (version: 1.28.3) [8] and TxDb.Btaurus.UCSC.bosTau9.refGene (version: 3.10.0).

### 2.11. Statistical Analysis

All experiments were conducted with a minimum of three biological and technical replicates. The graphical presentation and statistical analysis of the data were conducted using GraphPad Prism 8.0. The data are presented as means ± standard deviations (SDs). *p* < 0.05 was considered to indicate statistical significance.

## 3. Results

### 3.1. MBD3 KD Enhances the Ability of Bovine iPSCs to Differentiate into PGCLCs

Bovine fetal fibroblasts (BFFs) were reprogrammed into bovine iPSCs using the LCDM culture system [1]. The bovine iPSCs obtained by transfection with a nonsense sequence 48 h after reprogramming were iPSC-NC cells, while the bovine iPSCs obtained by transfection with MBD3 siRNA 48 h after reprogramming were iPSC-siMBD3 cells [9]. PGCLC differentiation was first conducted using 3D differentiation methods involving incipient mesoderm-like cells (iMeLCs) [2]. After 2 days of preinduction by Activin A and CHIR99021, bovine iPSCs were differentiated into iMeLCs. For 3D PGCLC induction, differentiated cells were maintained in floating culture conditions and aggregated into embryoid bodies in BMP4-, SCF-, EGF-, and LIF-containing medium (Figure S1A). The expression of PGC-related genes (*TFAP2C*, *PRDM1*, *SOX17*, *DAZL*, *DDX4*, and *NANOG*) in cells differentiated from iPSC-NC and iPSC-siMBD3 cells at the iMeLC stage and PGCLC stage was examined using qRT–PCR. The results revealed that the expression of *TFAP2C*, *SOX17*, *DAZL*, and *DDX4* was upregulated, while *PRDM1* expression in differentiated cells from the iPSC-siMBD3 and iPSC-NC groups and *DDX4* expression in differentiated cells from the iPSC-NC group were not upregulated (Figure S1B).

**Figure 1:**
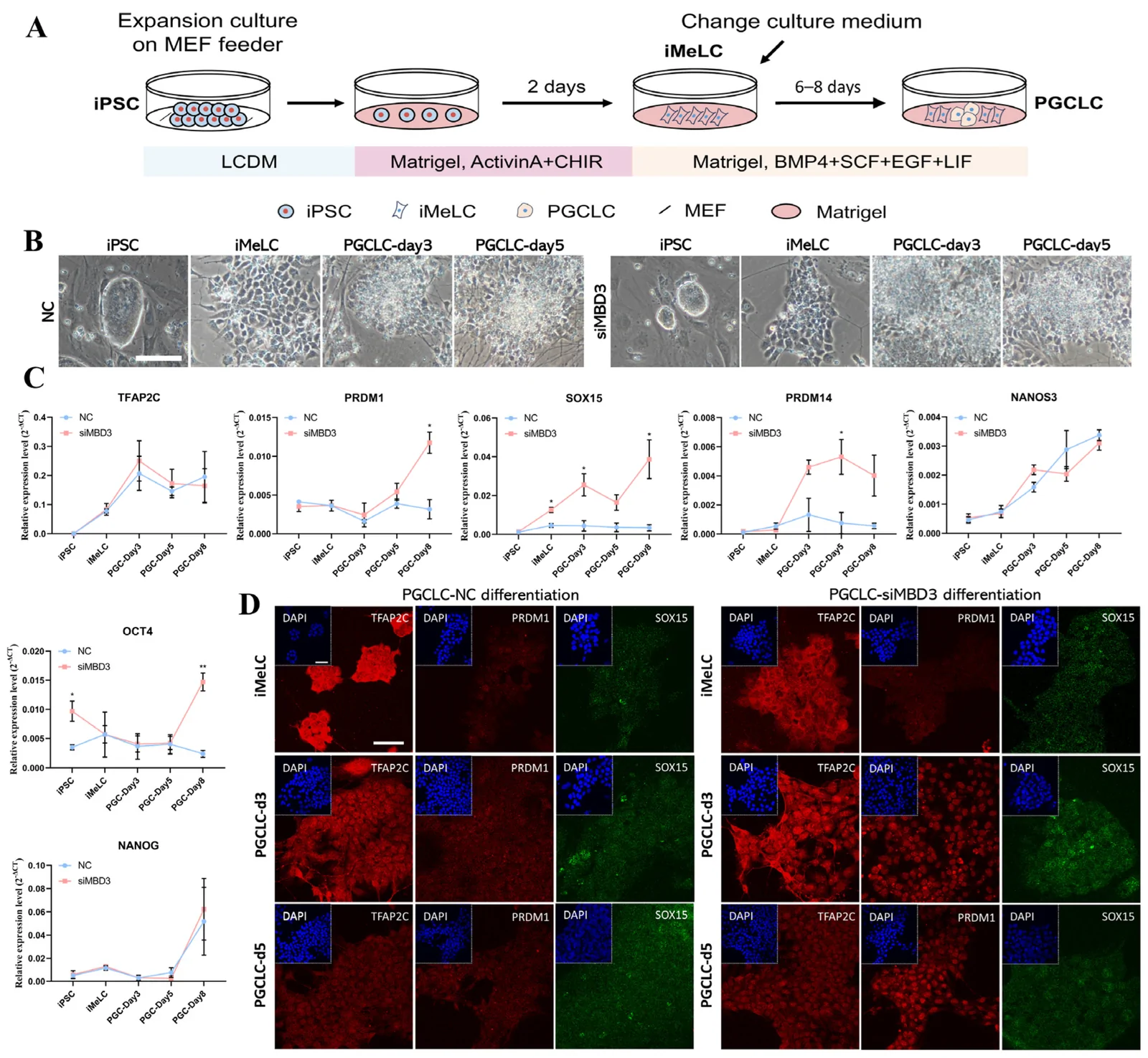
Generation of bovine PGCLCs from bovine iPSC-NC and iPSC-siMBD3 cells. (**A**) Diagram of bovine PGCLC induction by iPSC monolayer cell culture. (**B**) Images of the process of induced differentiation of bovine iPSCs into PGCLCs. Scale bars, 100 μm. (**C**) Expression of pluripotency and PGC marker genes during induction. * *p* < 0.5, ** *p* < 0.1. (**D**) Immunofluorescence staining of PGCLC markers (TFAP2C, PRDM1, and SOX15). Nuclei were stained with DAPI. Scale bars, 50 μm.

Since 3D differentiation methods were not efficient for obtaining bovine PGCLCs for subsequent mechanistic studies, we utilized a 2D method to generate bovine PGCLCs. iPSC-NC and iPSC-siMBD3 cells were first cultured on Matrigel with Activin A and CHIR99021 for 2 days to generate iMeLCs, after which the medium was changed to one containing BMP4, SCF, EGF, and LIF for PGCLC induction for 8 days (Figure 1A,B). To provide a comprehensive view of the germline specification trajectory, we analyzed the expression of PGC markers (*TFAP2C*, *PRDM1*, *SOX15*, *PRDM14*, NANOS3, OCT4 and *NANOG*) across the entire differentiation process, including the undifferentiated iPSC, pre-induced iMeLC, and PGCLC stages (Day 3, 5, and 8) (Figure 1C). SOX15 and SOX17 have been shown to be expressed in the PGCs of humans, macaques, pigs, goats, and bovines [10,11]. SOX15 is also highly expressed in early PGCs [12]. Upon PGCLC induction, PGCLCs initiated significant upregulation of the early PGC genes *TFAP2C* and *SOX15* and the pluripotency gene *NANOG* on day 3 and significant upregulation of *PRDM1* on day 5 (Figure 1C), which is similar to human PGCLC differentiation, in which TFAP2C is an upstream regulator of PRDM1 [13,14]. The expression of the *SOX17* gene began to increase on the third day of PGCLC differentiation (Figure S1C). Immunofluorescence staining revealed that TFAP2C was first synthesized in the cytoplasm during the preinduction stage and then entered the cell nucleus on the 3rd day of PGCLC induction to function as a transcription factor, and the protein expression level decreased on the 5th day. PRDM1 expression was low during the preinduction stage but began to increase on the 3rd day of PGCLC induction. However, the expression of PRDM1 was greater in the PGCLC-siMBD3 cells than in the PGCLC-NC cells on days 3 and 5. SOX15 began to accumulate on the 3rd day of differentiation, and partial nuclear expression was observed on the 5th day of differentiation. However, immunofluorescence staining revealed no nuclear expression of SOX15 in the PGCLC-NC cells on the 5th day of differentiation (Figure 1D). Moreover, SOX17 was not detected in the nuclei of PGCLCs during immunofluorescence staining (Figure S1D). The undifferentiated iPSC-NC and iPSC-siMBD3 cells were also stained using TFAP2C, PRDM1, and SOX15 antibodies, and a weak TFAP2C expression was identified (Figure S1E).

In addition, we modified the expansion and culture methods of marmoset PGCLCs and human PGCLCs and subcultured the obtained PGCLCs. Compared with that in PGCLCs, the expression of *TFAP2C* and *NANOG* in subcultured PGCLCs was reduced, the expression of *PRDM1* and *SOX17* was increased, and the expression of *SOX15* did not change significantly. Compared with PGCLC-NC cells, PGCLC-siMBD3 cells not only expressed higher levels of the *TFAP2C*, *SOX17*, and *SOX15* genes but also maintained the expression of these PGC marker genes in subcultured PGCLCs (Figure S2). In vitro embryoid body (EB) differentiation also confirmed that iPSC-siMBD3 cells expressed higher levels of *PRDM1* and lower levels of *OTX2* during differentiation (Figure S3). The latter is a transcription factor that hinders the differentiation of PGCLCs [15]. These results establish a 2D differentiation protocol for bovine iPSCs toward PGCLCs and identify the temporal sequence of *TFAP2C*/*SOX15* induction followed by *PRDM1* activation. *MBD3* knockdown was observed to enhance the expression of these key regulators during differentiation and sustain their expression during subsequent culture expansion.

**Figure 2:**
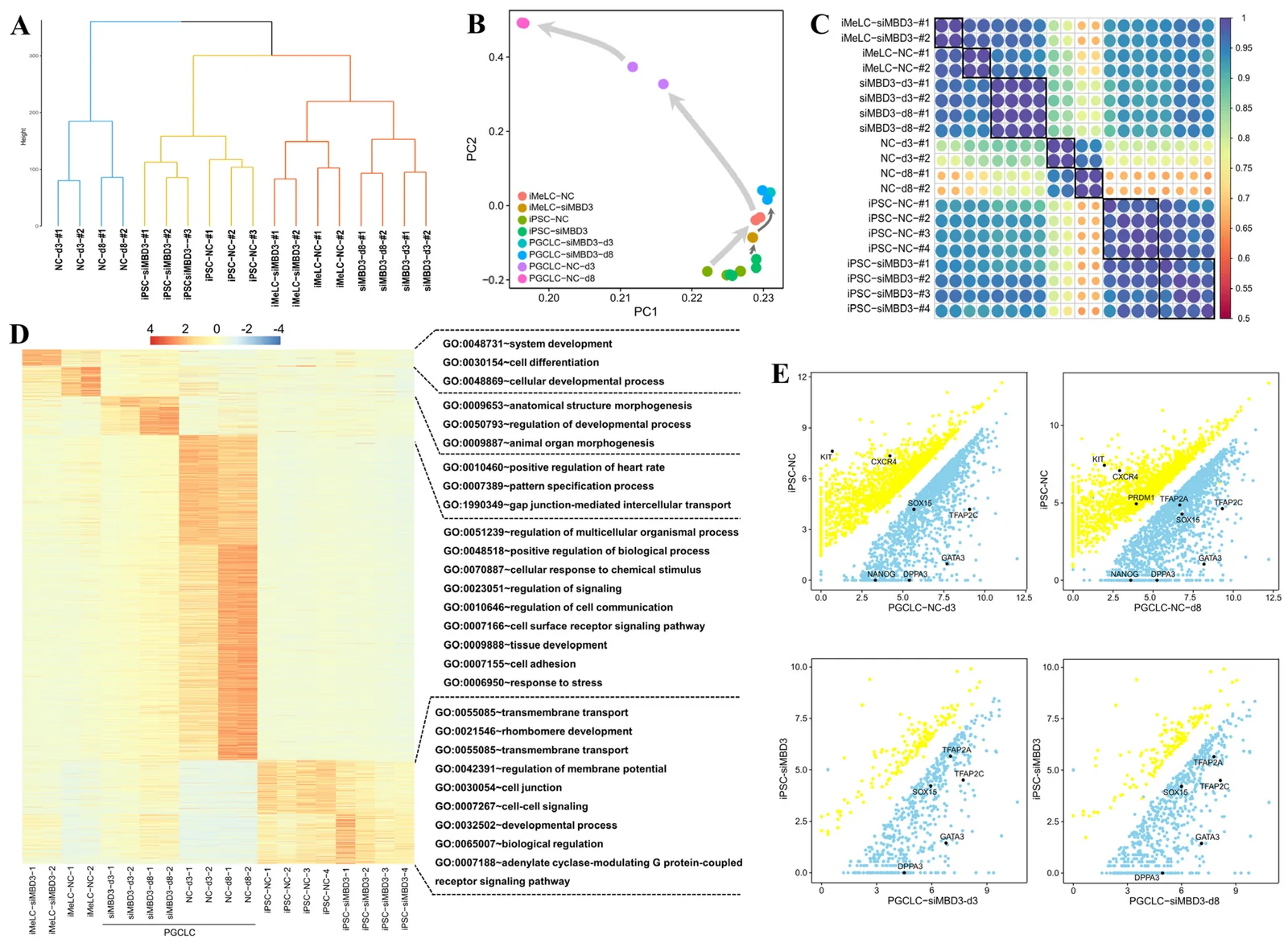
Transcriptome analyses of bovine PGCLCs. (**A**) UHC of the transcriptomes of bovine iPSCs, iMeLCs, and PGCLCs (d3 and d8). (**B**) PCA of bovine iPSCs, iMeLCs, and PGCLCs. (**C**) Pearson correlation between bovine iPSCs, iMeLCs, and PGCLCs. (**D**) Heatmap clustering of bovine iPSCs, iMeLCs, and PGCLCs (d3 and d8). The GO functional terms are shown for each gene cluster. (**E**) Scatter plot of genes upregulated (blue) or downregulated (yellow) in bovine PGCLCs compared with iPSCs (fold change > 4, FDR < 0.01).

To determine the global transcriptional differences between PGCLCs generated from iPSC-NC and iPSC-siMBD3 cells, RNA sequencing was conducted on days 0, 3, and 8 of differentiation. Unsupervised hierarchical clustering (UHC) first divided NC-d3, d8 cells, iPSCs, iMeLCs, siMBD3-d3, and siMBD3-d8 cells during the induction process of PGCLCs into two large clusters. The iPSCs, iMeLCs, siMBD3-d3, and siMBD3-d8 cells were divided into two clusters: iPSC-NC and iPSC-siMBD3 cells and siMBD3-d3 and d8 cells during iMeLC and PGCLC induction (Figure 2A). Principal component analysis (PCA), correlation coefficient analysis, and heatmaps consistently revealed differences in the directional and progressive transition pathways of cell properties during PGCLC induction of iPSC-NC and iPSC-siMBD3 cells (Figure 2B–D). The scatter plot analysis revealed that, compared with those in iPSCs, *TFAP2C*, *DPPA3*, *SOX15*, and *GATA3* were upregulated in PGCLCs. PGCLC-NC cells specifically upregulated *NANOG* expression, and this effect lasted until day 8, while PGCLC-siMBD3 cells specifically upregulated *TFAP2A* expression (Figure 2E). These results are consistent with those of a goat PGC analysis, which revealed that *SOX15* is highly expressed in early PGCs, whereas *TFAP2A* and *GATA3* are highly expressed in the PGCs of male goats [10]. Moreover, SOX15 is among the transcription factors that can replace SOX2 in human PGCs [16], which may explain why SOX2 is suppressed in human, macaque, pig, goat and bovine PGCs [10,11]. The cell state transition process differed between iPSC-NC and iPSC-siMBD3 cells during PGCLC differentiation: compared with those in iPSC-NC cells, the numbers of upregulated or downregulated genes in iPSC-siMBD3 cells and the cell state transition during the induction of PGCLCs were much lower (Figure S4).

### 3.2. scRNA-Seq Indicates That Bovine PGCLCs with MBD3 KD Are More Similar to In Vivo PGCs

**Figure 3:**
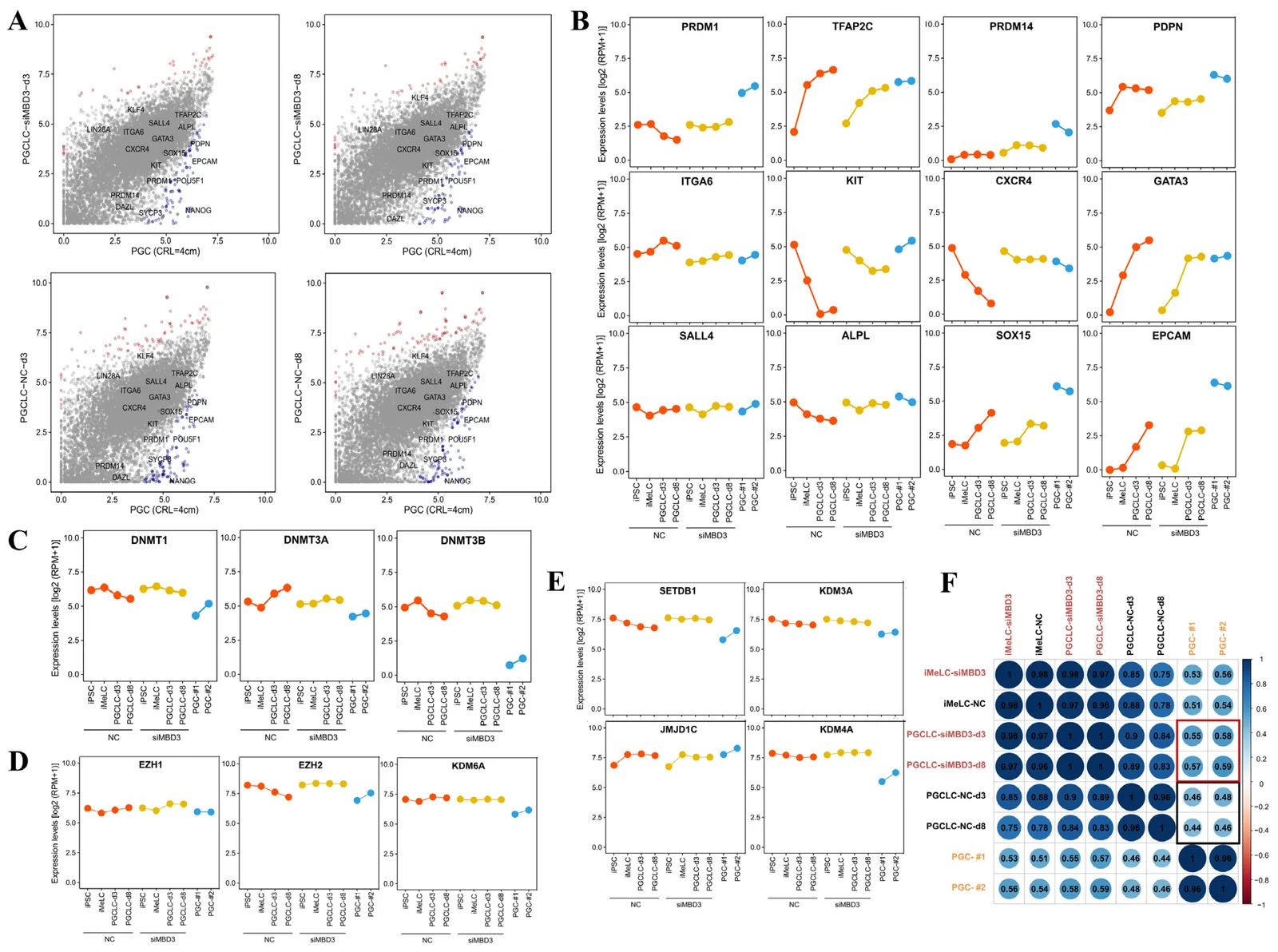
Transcriptome analysis showing that bovine PGCLCs with MBD3 KD are similar to PGCs in vivo (CRL = 4 cm). (**A**) Comparison of the transcriptomes of bovine PGCLCs at 3 and 8 days of differentiation (top: PGCLC-siMBD3 cells; bottom: PGCLC-NC cells) and PGCs. Scatter plots showing the transcriptomes of cells with DEGs. (**B**) Comparison of the expression of genes involved in PGCLC differentiation between PGCLCs and PGCs. Each value and the average gene expression based on transcriptome analysis using biological replicates are shown. (**C**) Comparison of the expression of genes involved in DNA methylation between PGCLCs and PGCs. Each value and average gene expression based on transcriptome analysis using biological replicates are shown. (**D**) Comparison of the expression of genes associated with H3K27me3 between PGCLCs and PGCs. Each value and average gene expression based on transcriptome analysis using biological replicates are shown. (**E**) Comparison of H3K9me3 gene expression between PGCLCs and PGCs. Each value and average gene expression based on transcriptome analysis using biological replicates are shown. (**F**) Pearson correlation between bovine iPSC-NC and iPSC-siMBD3 cell-derivative iMeLCs, PGCLCs, and PGCs in vivo.

Before we performed scRNA-seq, we compared the bulk RNA-seq data of PGCLC-NC cells, PGCLC-siMBD3 cells, and bovine PGCs at early developmental stages (CRL = 4 cm) [11]. There were fewer differentially expressed genes (DEGs) between PGCLC-siMBD3 cells and PGCs than between PGCLC-NC cells and PGCs (Figure 3A). The expression levels of many key genes, such as *PRDM1*, *TFAP2C*, *ITGA6*, *KIT*, *CXCR4*, *GATA3*, *SALL4*, and *ALPL*, in PGCLC-siMBD3 cells were similar to those in bovine PGCs (Figure 3B). The mRNA expression levels of DNA methylation- and histone methylation-related enzymes in PGCLC-siMBD3 cells were also very similar to those in bovine PGCs, except for those of *DNMT3B* (Figure 3C–E). The correlation coefficient between PGCLCs from iPSC-siMBD3 cells and PGCs in fetal bovine gonads was greater than that between PGCLCs from iPSC-NC cells and PGCs in fetal bovine gonads (Figure 3F). In addition, we compared the transcriptomes of PGCLCs with those of goat and porcine PGCs [10]. PCA using all expressed genes revealed that the goat, porcine, and bovine profiles formed clusters within each species. The cells differentiated in a similar direction along PC3 according to the order of differentiation, and the PGCLC-siMBD3 cells were closer to the goat and pig PGCs in the PC1 direction (Figure S5A,C). A comparison of bovine PGCLCs with human and mouse PGCLCs revealed that PGCLC-siMBD3 cells were similar to human and mouse PGCLCs in the PC2 direction (Figure S5B,D).

**Figure 4:**
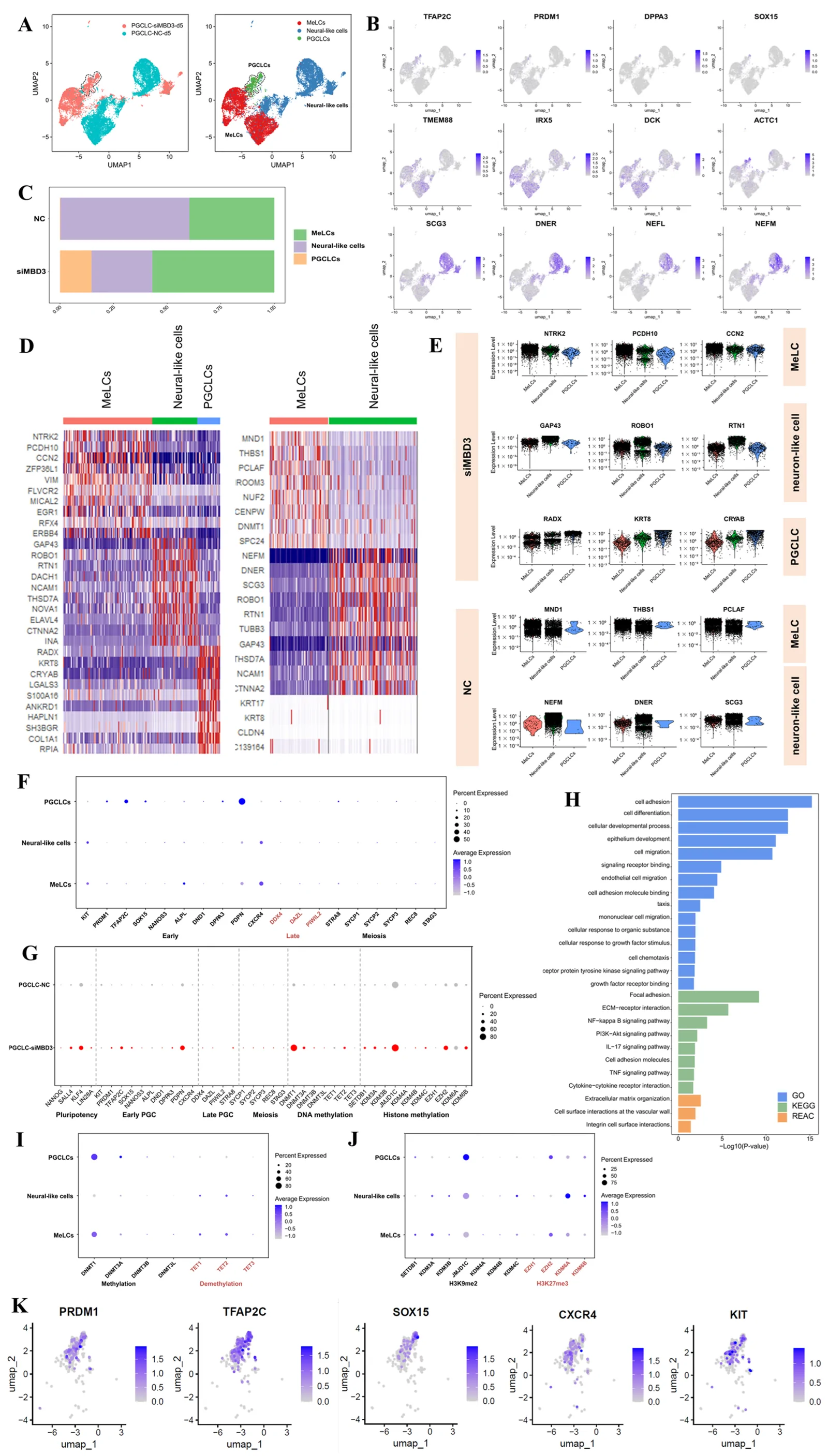
Single-cell transcriptome analysis of PGCLCs at differentiation day 5. (**A**) Uniform manifold approximation and projection (UMAP) plot showing different cell types in the PGCLC-NC and PGCLC-siMBD3 groups on day 5 of differentiation. Cell clusters are annotated on the basis of marker genes. Clusters representing bovine PGCLCs are circled. (**B**) Expression levels of PGCLCs, MeLCs, and neuronal-like cell markers in bovine PGCLC-NC and PGCLC-siMBD3 cells. (**C**) Proportions of PGCLCs, MeLCs, and neuronal-like cells among bovine PGCLC-NC and PGCLC-siMBD3 cells. (**D**) Heatmap of DEGs in each cell cluster in PGCLC-siMBD3 cells (left) and PGCLC-NC cells (right). (**E**) Genes highly expressed in the MeLC cluster, neuron-like cell cluster, and PGCLC cluster in PGCLC-siMBD3 cells and PGCLC-NC cells are shown as violin plots of log-normalized expression. (**F**) The proportion of cells expressing early PGCs, late PGCs, and meiotic markers in each cell cluster of PGCLC-siMBD3 cells. (**G**) Proportions of cells expressing markers of pluripotency, early PGCs, late PGCs, meiosis, DNA methylation, and histone methylation in bovine PGCLC-NC and PGCLC-siMBD3 cells. (**H**) GO enrichment analysis, Kyoto Encyclopedia of Genes and Genomes, and Reactome enrichment analysis were performed on genes that were significantly more highly expressed in the PGCLC cluster in PGCLC-siMBD3 cells. (**I**) Dot plots showing the expression of DNA methylation- and DNA demethylation-related genes in different cell clusters in bovine PGCLC-siMBD3 cells. (**J**) Dot plots showing the expression of H3K9me2- and H3K27me3-related genes in different cell clusters in bovine PGCLC-siMBD3 cells. (**K**) Feature plots with expression of early germline (*PRDM1*, *TFAP2C*, *SOX15*) and migration (*CXCR4*, *KIT*) genes in bovine PGCLCs.

To further confirm the production of bovine PGCLCs from iPSC-NC and iPSC-siMBD3 cells, differentiated cells on day 5 were subjected to scRNA-seq. Three cell clusters were identified, in which cells expressing the germline-specific genes *TFAP2C*, *PRDM1*, *DPPA3*, and *SOX15* and the pluripotency gene were identified as PGCLCs; cells expressing *TMEM88*, *IRX5*, *DCK*, and *ACTC1* were identified as mesenchymal-like cells (MeLCs); and cells expressing *SCG3*, *DNER*, *NEFL*, and *NEFM* were identified as neural-like cells (Figure 4A,B). There were almost no PGCLCs in differentiated iPSC-NC cells (28 out of 14,054 total cells, 0.20%), whereas differentiated iPSC-siMBD3 cells contained a substantial proportion of PGCLCs (1354 out of 9203 total cells, 14.71%). Most of the cells differentiated from iPSC-NC cells were neural-like cells (Figure 4C and Figure S6A,B). The iPSC-siMBD3 cell-derived PGCLC cluster also highly expressed genes such as *RADX*, *KRT8*, and *CRYAB*, which are associated with a stable genome and cell structure (Figure 4D,E). Among them, KRT8 is highly expressed in PGCLCs and plays a crucial role in germ cell migration [17]. The iPSC-siMBD3 cell-derived PGCLC clusters differentially expressed the primordial germ cell markers, with strong *PDPN*, moderate *PRDM1*, and detectable *TFAP2C* and *SOX15* expression, whereas the MeLC clusters and neural-like cell clusters did not express these genes or expressed them at very low levels (Figure 4F,G).

The iPSC-siMBD3 cell-derived PGCLC clusters did not express late primordial germ cell- or meiosis-related marker genes such as *DAZL*, *DDX4*, or *SYCP1/2/3* (Figure 4F,G). Gene Ontology (GO) enrichment analysis was performed on the genes expressed in the PGCLC cluster, and enrichment in functions such as “cell differentiation”, “cell development process”, and “cell migration” was detected (Figure 4H). In addition, the PGCLC cluster highly expressed the H3K9me2 demethylase *JMJD1C* and the H3K27me3 methylase *EZH2*, as well as the DNA methyltransferase *DNMT1* (Figure 4I,J). The scRNA-seq data revealed that most cells in the PGCLC cluster expressed the DNA methyltransferase *DNMT1* (76%), the H3K9me2 demethylase *JMJD1C* (83.2%), and the H3K27me3 methylase *EZH2* (45.1%) (Table 1). Bulk RNA-seq showed that PGCLC-siMBD3 cells clustered more closely with PGC transcriptomes from multiple species than PGCLC-NC cells. scRNA-seq further revealed that only iPSC-siMBD3 cultures generated a distinct PGCLC cluster at differentiation day 5, while iPSC-NC cells predominantly differentiated into neural-like cells. Within the iPSC-siMBD3 PGCLC cluster (1354 cells), the majority of cells expressed *PDPN* (50.37%, 682/1354), *TFAP2C* (25.18%, 341/1354), *PRDM1* (9.74%, 132/1354), or *SOX15* (10.49%, 142/1354) (Table 2). Notably, 23 cells (1.70%) co-expressed all four canonical PGCLC markers (*TFAP2C+*, *PRDM1+*, *SOX15+*, and *PDPN+*), representing a highly purified PGCLC subpopulation (Figure 4K). The siMBD3-PGCLCs expressed genome-stabilizing genes (*KRT8*, *RADX*, and *CRYAB*) and epigenetic modifiers (*JMJD1C* and *EZH2*) but lacked late/meiotic markers, consistent with an early PGCLC state.

### 3.3. MBD3 KD Promotes PRDM1 and SOX15 Expression in Bovine PGCLCs by Increasing NODAL Signaling in the TGF-β Superfamily

**Figure 5:**
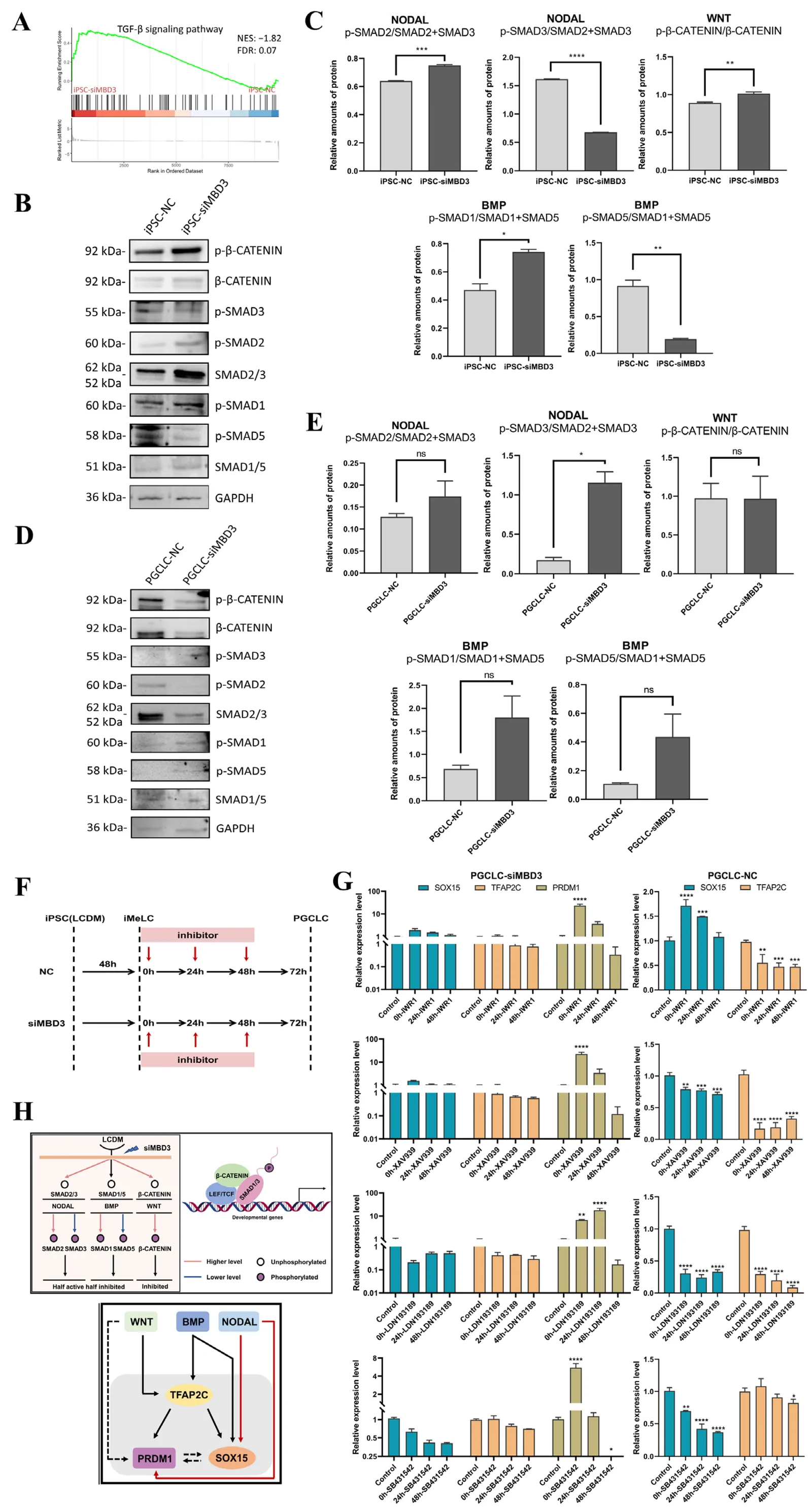
Function of NODAL, BMP, and WNT signaling in bovine iPSCs and PGCLCs. (**A**) GSEA of iPSC-NC and iPSC-siMBD3 cells. (**B**) Expression of key proteins of the NODAL, BMP, and WNT signaling pathways in bovine iPSCs. (**C**) Quantitative density analysis of the proteins in (**B**). * *p* < 0.5, ** *p* < 0.1, *** *p* < 0.01, **** *p* < 0.001. (**D**) Expression of key proteins in the NODAL, BMP, and WNT signaling pathways in PGCLCs. (**E**) Quantitative density analysis of the proteins in (**D**). * *p* < 0.5. (**F**) Time points at which signaling pathway inhibitors were added. (**G**) Changes in PGC marker gene expression after the addition of signaling pathway inhibitors. (**H**) Schematic diagram of the functions of NODAL, BMP, and WNT in bovine PGC differentiation. Solid lines indicate experimentally observed relationships; dashed lines indicate relationships that remain to be experimentally validated; red lines indicate enhanced effects observed in PGCLC-siMBD3 cells.

BMP, NODAL, and WNT signaling are important for PGC specification. BMP and NODAL signaling can activate the expression of *TFAP2C* and *SOX17*, whereas WNT signaling is transient and is responsible for the induction of NODAL [2,13,18]. Once WNT signaling is activated during differentiation, β-CATENIN and R-SMAD (p-SMAD1 or p-SMAD3) bind to developmental genes or genes related to cell fate determination to regulate transcription [19], and NODAL and BMP are subsequently activated to participate in the regulation of differentiation [13]. We next asked whether there is a difference in the signal transduction of these three key pathways during bovine PGCLC differentiation between iPSC-NC and iPSC-siMBD3 cells. To answer this question, we first performed gene set enrichment analysis on the transcriptome data of iPSC-NC and iPSC-siMBD3 cells, and the results revealed that the expression of genes involved in the “TGF-β signaling pathway” in iPSC-siMBD3 cells was significantly increased compared with that in iPSC-NC cells (Figure 5A). Western blot analysis revealed that, compared with iPSC-NC cells, iPSC-siMBD3 cells expressed high levels of β-CATENIN and p-β-CATENIN protein. The total protein levels of the effectors (SMAD2 and SMAD3) of NODAL signaling increased significantly, the p-SMAD2/SMAD2 + SMAD3 ratio increased, and the p-SMAD3/SMAD2 + SMAD3 ratio decreased, indicating that, although the NODAL signal in iPSC-siMBD3 cells was in a semiactivated state, the expression of the substrates SMAD2 and SMAD3 was high. The total protein levels of the effectors (SMAD1 and SMAD5) of BMP signaling were similar, the p-SMAD1/SMAD1 + SMAD5 ratio increased, and the p-SMAD5/SMAD1 + SMAD5 ratio decreased, indicating that BMP signaling in iPSC-siMBD3 cells was also activated (Figure 5B,C). In summary, in iPSC-siMBD3 cells, WNT signaling is maintained at a low basal level, while BMP and NODAL signaling exhibits a coordinated activation with distinct phosphorylation patterns for their respective SMAD effectors. Specifically, NODAL signaling displays elevated total SMAD2/3 protein with increased p-SMAD2 phosphorylation, while BMP signaling shows enhanced p-SMAD1 activity despite stable total SMAD1/5 levels.

Many effects of BMP4 are known to be mediated indirectly through the BMP-WNT-NODAL cascade [13]. To test the effects of the three signaling pathways on the induction of bovine PGCLCs, we detected the expression of their key proteins during the differentiation process. Compared with those in the PGCLC-NC cells, the protein levels of β-CATENIN and p-β-CATENIN were decreased in the PGCLC-siMBD3 cells. The total protein levels of the effectors (SMAD2 and SMAD3) of NODAL signaling were decreased, and the p-SMAD2 protein level was decreased, whereas the p-SMAD3 protein level was increased. The total protein levels of the effectors (SMAD1 and SMAD5) of BMP signaling were similar, and the p-SMAD1 and p-SMAD5 protein levels were increased (Figure 5D,E). These results indicate that iPSC-siMBD3 cells are more dependent on BMP and NODAL signaling during differentiation into PGCLCs.

To verify this hypothesis, we applied the BMP receptor inhibitor LDN193189 (250 nM), the NODAL inhibitor SB431542 (10 μM), or the WNT inhibitors IWR1 (5 μM) and XAV939 (5 μM) at 0 h, 24 h, or 48 h of the iMeLC stage (Figure 5F) and analyzed gene expression at the terminal stage of PGCLC differentiation (72 h). In PGCLC-NC cells, BMP inhibition (LDN193189) decreased both *SOX15* and *TFAP2C*, WNT inhibition (IWR1 or XAV939) decreased *TFAP2C*, and NODAL inhibition (SB431542) decreased *SOX15*, indicating that *TFAP2C* induction depends on BMP and WNT signaling, whereas *SOX15* induction requires BMP and NODAL signaling (Figure 5G). Notably, any of these three inhibitions abolished *PRDM1* expression in PGCLC-NC cells at the terminal stage, demonstrating that wild-type *PRDM1* expression relies on the integrated activity of BMP, NODAL, and WNT signaling. In stark contrast, in PGCLC-siMBD3 cells, only NODAL inhibition (SB431542) eliminated PRDM1 at the terminal stage, whereas BMP or WNT inhibition failed to do so (Figure 5G), indicating that *MBD3* knockdown shifts *PRDM1* regulation from multi-pathway dependency to NODAL-dominant control (Figure 5H).

These terminal-stage lineage-specification effects were distinct from the transient responses observed upon early inhibition. When applied at 0 h or 24 h of the iMeLC stage, LDN193189 caused a robust increase in *PRDM1* expression at the terminal stage of PGCLC differentiation in siMBD3 cells (Figure 5G), suggesting a compensatory upregulation upon early BMP withdrawal. Similarly, SB431542 applied at 0 h induced a transient increase in *PRDM1* that was not sustained when the inhibitor was added at 24 h or 48 h (Figure 5G), indicating the existence of a narrow early window during which signaling disruption triggers a feedback mechanism that transiently activates *PRDM1*. In addition, the inhibition of either pathway at 0 h or 24 h promoted *SOX17* expression during PGCLC differentiation (Figure S7B).

*MBD3* knockdown altered the signaling requirements for PGCLC specification: *TFAP2C* and *SOX15* maintained their dependence on BMP/WNT and BMP/NODAL signaling, respectively, while *PRDM1* expression shifted from multi-pathway control to primarily NODAL-dependent regulation at the lineage-specification stage. Notably, transient feedback activation of *PRDM1* was observed upon early BMP or NODAL withdrawal.

### 3.4. MBD3 KD Promotes the Differentiation of Bovine iPSCs into PGCLCs Through the Addition of Bivalent Modification of TGF-β-Related Genes

**Figure 6:**
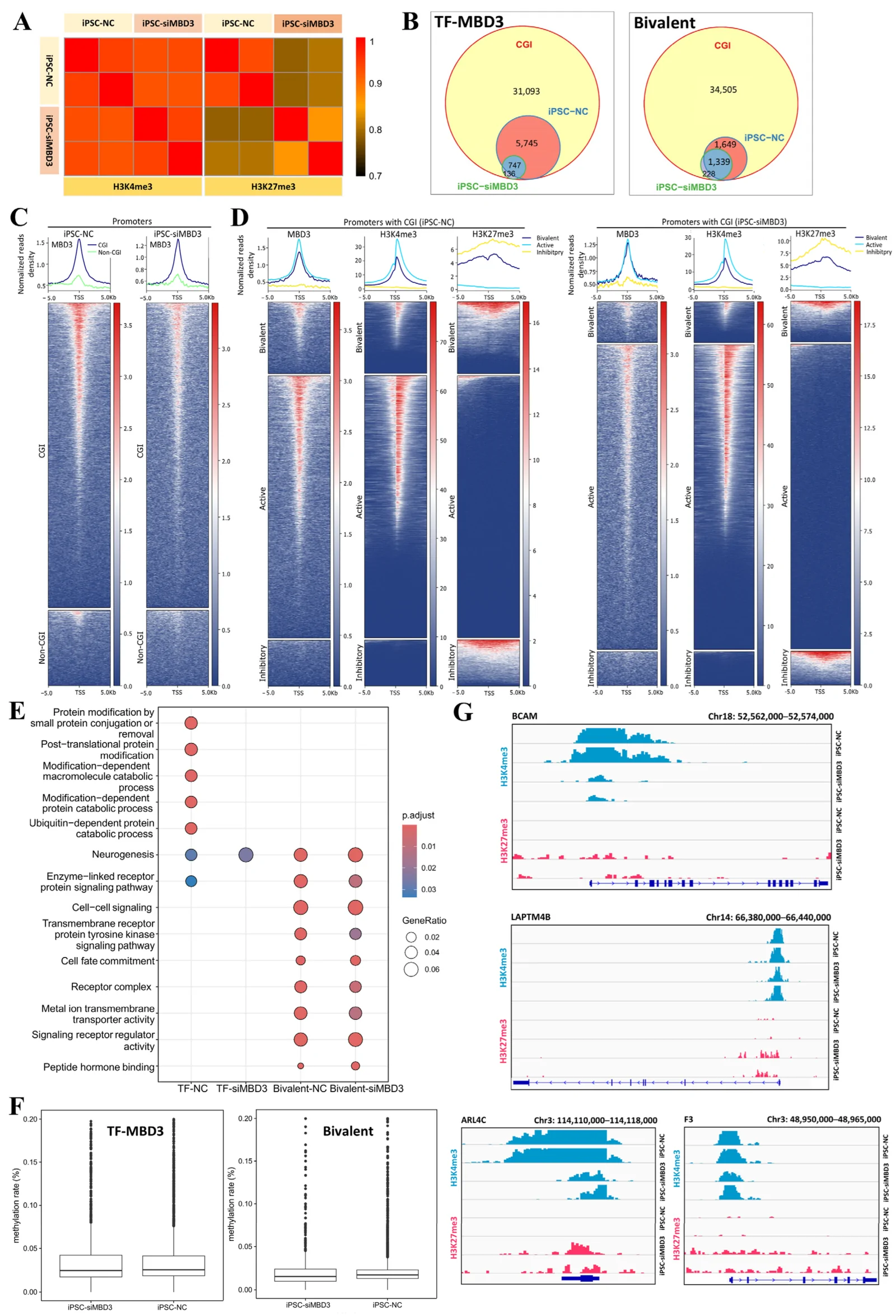
Comparison of H3K4me3, H3K27me3, and bivalent modification genes in bovine iPSC-NC and iPSC-siMBD3 cells. (**A**) Heatmap representation (correlation coefficient) of changes in H3K4me3 and H3K27me3 in bovine iPSC-NC and iPSC-siMBD3 cells. (**B**) Venn diagram showing the overlap of binding regions of MBD3 (**left**) or bivalent modifications (**right**) on CGIs in iPSC-NC and iPSC-siMBD3 cells. (**C**) Heatmaps of MBD3 ChIP-seq signals around the TSSs of CGI and non-CGI promoters. (**D**) Heatmaps of MBD3 ChIP-seq signals around the TSSs of different types of CGI promoters. (**E**) GO annotation results of genes overlapping with MBD3 or bivalent modification binding regions on CGIs in iPSC-NC and iPSC-siMBD3 cells. (**F**) Average relative (percent) CpG methylation levels in regions bound by MBD3 (**top**) or bivalent modifications (**bottom**) in iPSC-NC and iPSC-siMBD3 cells. (**G**) Increased bivalent genes in iPSC-siMBD3 cells.

To further investigate how *MBD3* knockdown potentiates PGCLC fate, genome-wide maps of MBD3, H3K4me3, and H3K27me3 were generated using ChIP-seq for both iPSC-NC and iPSC-siMBD3 cells. The results revealed that the global changes in H3K27me3 were more obvious than those in H3K4me3 (Figure 6A). The number of genes specifically bound by H3K4me3 in iPSC-siMBD3 cells was similar to that in iPSC-NC cells (iPSC-siMBD3 cells: 405; iPSC-NC cells: 434). However, the number of genes specifically bound by H3K27me3 in iPSC-siMBD3 cells significantly differed from that in iPSC-NC cells (iPSC-siMBD3 cells: 288; iPSC-NC cells: 1517) (Figure S8A). We identified genes bound by MBD3 on CGIs and promoters in both cell lines and reported that the number of genes bound by MBD3 on CGIs or promoters was significantly reduced in iPSC-siMBD3 cells (Figure 6C). These findings also confirmed that the effects of MBD3 KD at the early stage of reprogramming can be maintained in established iPSC lines. A total of 1339 bivalently marked CGI-associated genes were found to be common to both cell lines, and an additional 228 genes were detected to carry bivalent modifications exclusively in the iPSC-siMBD3 cells. Furthermore, the addition of these bivalent modifications was closely associated with H3K27me3 (Figure 6B,D). To explore how *MBD3* KD affects the differentiation of iPSCs into PGCLCs through bivalent modification, the functions and methylation levels of bivalent genes shared by iPSC-siMBD3 and iPSC-NC cells were analyzed. The results revealed that the bivalent genes shared by iPSC-siMBD3 and iPSC-NC cells had similar biological functions and similar methylation levels (Figure 6E,F), which indicates that *MBD3* KD may promote the differentiation of PGCLCs by increasing the bivalent modification of certain genes. Therefore, the specific bivalent genes in iPSC-siMBD3 cells were analyzed, and the results revealed that most of these genes were related to the TGF-β superfamily, which has been shown to play a role in testicular development, inhibiting proliferation and promoting the migration of PGCs to gonadal ridge explants in vitro [20,21,22,23,24], with representative genes including *ARL4C* [25], *BCAM* [26], *LAPTM4B* [27], and *F3* [28] (Figure 6G). *MBD3* knockdown was associated with increased bivalent H3K4me3/H3K27me3 modifications at promoters of TGF-β-related genes during PGCLC differentiation.

### 3.5. MBD3 KD Increases the Expression of DPPA4 and DNMT3L via HDAC1 to Alter Bivalent Chromatin Modification in Bovine iPSCs

**Figure 7:**
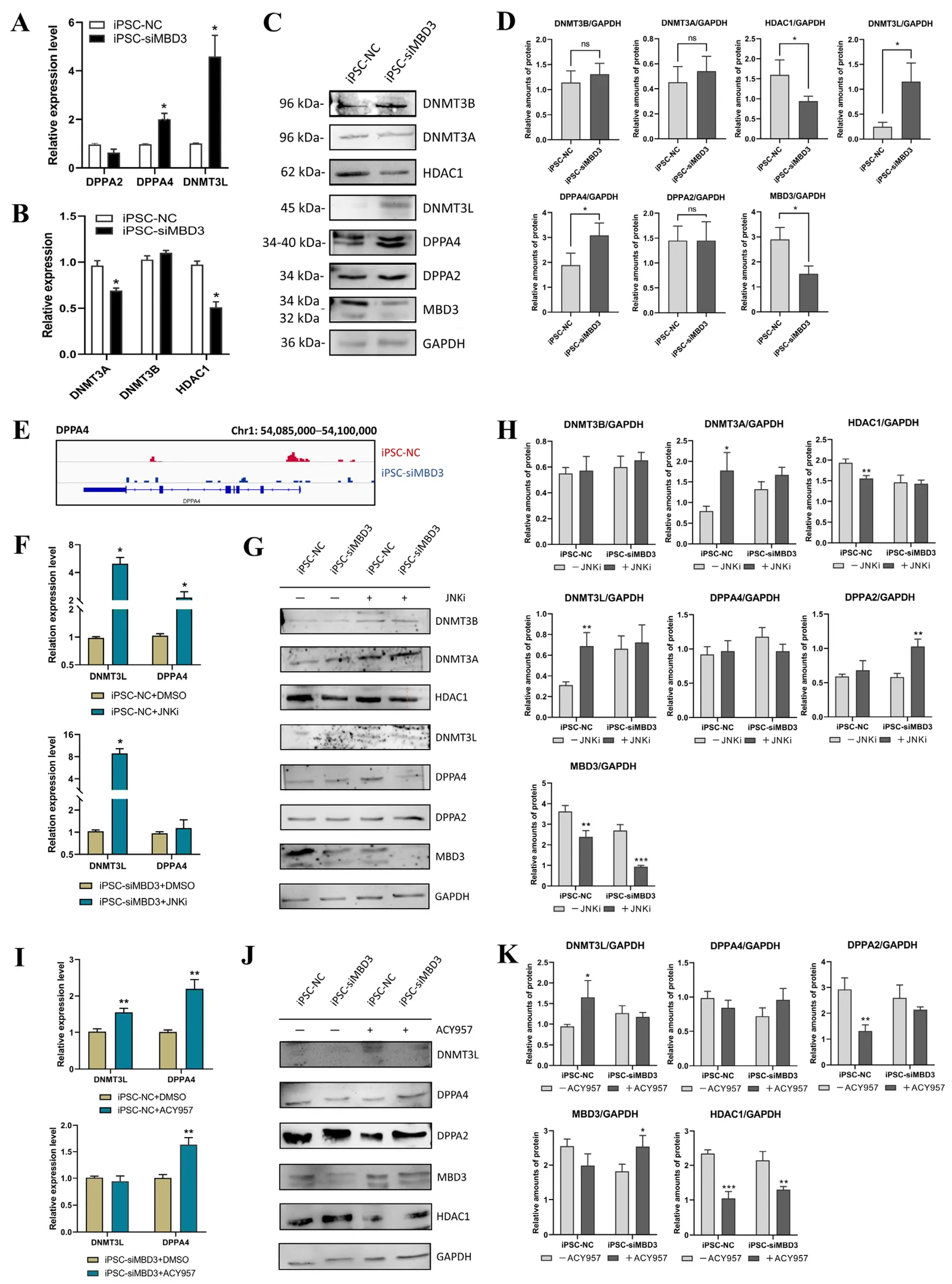
DNMT3L and DPPA4 modify bivalent promoters. (**A**) Expression of *DPPA2/4* and *DNMT3L* in bovine iPSCs. * *p* < 0.5. (**B**) Expression of *DNMT3A/B* and *HDAC1* in bovine iPSCs. * *p* < 0.5. (**C**) Total MBD3, HDAC1, DPPA2/4, and DNMT3L/A/B protein levels. (**D**) Densitometric analysis of the protein quantification in (**C**). * *p* < 0.5. (**E**) ChIP-seq reveals MBD3 binding in a 15 kb region surrounding *DPPA4*. (**F**) Expression levels of *DPPA4* after the addition of a JNK inhibitor. * *p* < 0.5. (**G**) Changes in the protein levels of MBD3, HDAC1, DPPA2/4, and DNMT3L/A/B after the addition of a JNK inhibitor. (**H**) Densitometric analysis of the protein quantification in (**G**). * *p* < 0.5, ** *p* < 0.1. (**I**) Expression levels of *DPPA4* after the addition of an HDAC1 inhibitor. ** *p* < 0.1. (**J**) Changes in the protein levels of MBD3, HDAC1, DPPA2/4, and DNMT3L after the addition of an HDAC1 inhibitor. (**K**) Densitometric analysis of the protein quantification in (**J**). * *p* < 0.5, ** *p* < 0.1, *** *p* < 0.01.

Previous studies have shown that DNMT3L interacts with PRC2 and competes with the DNA methyltransferases DNMT3A and DNMT3B to maintain low methylation levels in H3K27me3 regions of bivalent genes [29], whereas DPPA2/4 maintains low methylation in H3K4me3 regions in bivalent genes [30]. In addition, our previous studies demonstrated that DPPA2/4 could increase the differentiation potential of bovine extended pluripotent stem cells (EPSCs) [31]. To explore the mechanism through which MBD3 affects bivalent modification, changes in DPPA4 and DNMT3L were investigated. The results of qRT–PCR revealed that the expression of *DPPA4* and *DNMT3L* in iPSC-siMBD3 cells was greater than that in iPSC-NC cells, whereas the expression of *DPPA2* was not significantly different (Figure 7A).

Given that MBD3, as a member of the NuRD complex, is associated not only with histone acetylation but also with DNA methylation and can recruit DNMT3A/B to inhibit target genes in gene regulation [32], the expression of DNMT3A/B and HDAC1/2 in iPSC-siMBD3 and iPSC-NC cells was investigated. The mRNA level of *DNMT3A* in the iPSC-siMBD3 cells was significantly lower than that in the iPSC-NC cells, and the level of *DNMT3B* was similar; however, the protein expression levels of DNMT3A in the two cell lines were similar, while the level of DNMT3B in the iPSC-siMBD3 cells was greater than that in the iPSC-NC cells. In addition, the mRNA and protein levels of HDAC1 in iPSC-siMBD3 cells were significantly reduced (Figure 7B–D). ChIP-seq revealed that MBD3 had a direct regulatory effect on *DPPA4*, but not on *DPPA2* or *DNMT3L* (Figure 7E). Therefore, we hypothesized that MBD3 can directly regulate the expression of the *DPPA4* gene by recruiting DNMT3A/B or HDAC1, and that MBD3 may indirectly regulate the expression of DNMT3L through HDAC1.

To test our hypothesis, we attempted to detect changes in DPPA4 and DNMT3L by inhibiting the expression of MBD3. Owing to the short-term effects of siRNA, observing the interactions between proteins and genes or between proteins and proteins may be impossible. Therefore, we tried to use activators or inhibitors of factors that interact with MBD3. MBD3 interacts with the c-Jun transcriptional activation domain, which leads to reduced target gene expression through the recruitment of the NuRD repressor complex. The phosphorylation of c-Jun by Jun N-terminal kinases (JNKs) can increase target gene transcription [33,34]. However, whether the addition of JNK inhibitors promotes or inhibits MBD3 is currently unknown. To confirm the effect of the JNK inhibitor on the expression of MBD3, iPSC-siMBD3 and iPSC-NC cells were treated with the JNK inhibitor SP600125, which resulted in a significant decrease in MBD3 protein expression. After iPSC-NC cells were treated with SP600125, the reduction in MBD3 protein expression led to a reduction in HDAC1 protein expression, thereby promoting the expression of the *DPPA4* gene while increasing the expression of DNMT3L protein. Since the number of MBD3 protein-bound genes in iPSC-siMBD3 cells was significantly reduced, the expression of the HDAC1 protein was significantly reduced, and the MBD3 protein could no longer regulate the expression of many genes, including *DPPA4*; thus, the reduction in MBD3 protein expression in iPSC-siMBD3 cells no longer affected the expression of the HDAC1 protein and thereby failed to affect the expression of the DNMT3L protein (Figure 7F–H). Furthermore, the reduction in MBD3 protein expression led to an increase in DNMT3A and DNMT3B protein expression, possibly because feedback regulation increased the function of MBD2/NuRD.

**Figure 8:**
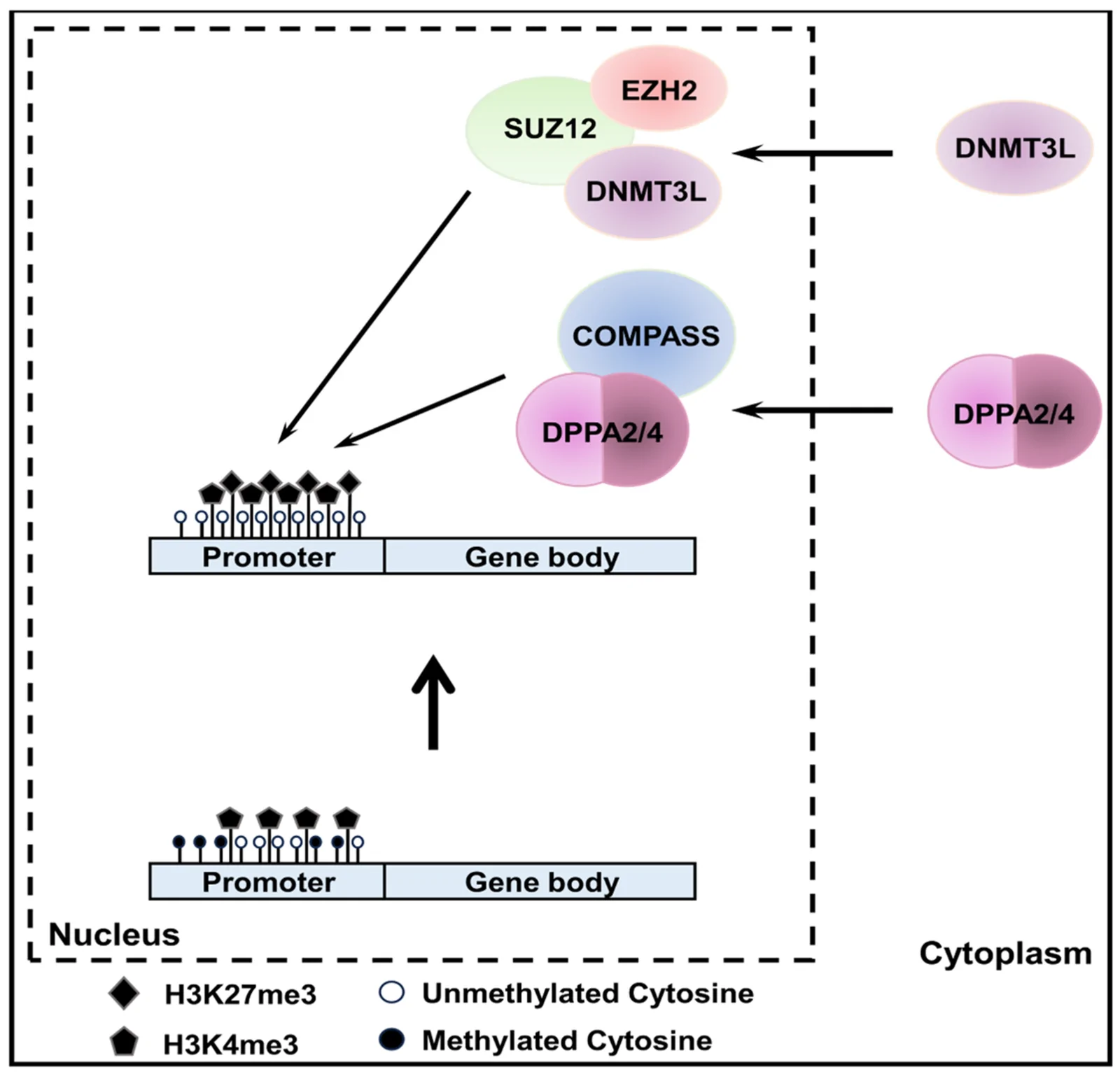
Mechanism through which DNMT3L and DPPA4 modify bivalent promoters. A reduction in MBD3 protein expression increases the expression of DPPA4 and DNMT3L. DPPA4 binds to the promoter marked by H3K4me3 and stabilizes the H3K4me3 level, whereas DNMT3L binds to the promoter marked by H3K4me3, which is bound by DPPA4, and establishes the H3K27me3 mark.

DNMT family members have a common and conserved feature in that they are able to recruit HDAC1 to inhibit gene transcription, including DNMT3L, which has no methyltransferase domain but retains the PHD domain to contact HDAC1 [35,36,37]. To confirm the functional relationship between DNMT3L and HDAC1 in iPSC-siMBD3, we treated iPSCs with ACY-957, an HDAC1 inhibitor. As expected, the inhibition of HDAC1 in iPSC-NC cells increased *DPPA4* gene expression and DNMT3L protein levels (Figure 7I–K). These results suggest that DNMT3L interacts with HDAC1 in iPSC-NC cells, and that the pharmacological inhibition of HDAC1 is associated with increased DNMT3L protein levels. Whether this reflects a direct compensatory feedback loop or an indirect effect of altered chromatin state remains to be determined. Notably, a reduction in HDAC1 expression does not lead to a decrease in MBD3 protein levels, but a reduction in MBD3 expression can reduce HDAC1 expression, suggesting that MBD3 plays a decisive role in binding to HDAC1. In conclusion, the knockdown of *MBD3* increases the expression of DPPA4 and DNMT3L. DPPA4 binds to the promoter marked by H3K4me3, stabilizes the H3K4me3 level, and prevents de novo DNA methylation. DPPA4 inhibits the binding of HDAC1 and promotes the recruitment of DNMT3L to the H3K4me3-marked promoter. DNMT3L forms a complex with PRC2 components (EZH2 and SUZ12) at these promoters and facilitates PRC2-mediated establishment of the H3K27me3 mark, whereas DPPA4–DNMT3L cooperatively prevents de novo DNA methylation (Figure 8). In this way, the promoter of development-related genes is modified in a bivalent manner.

## 4. Discussion

MBD3 is a critical rate-limiting barrier in the process of somatic cell reprogramming. The depletion of MBD3 results in the deterministic and synchronous reprogramming of iPSCs [38], and our previous study revealed that the knockdown of MBD3 promotes the reprogramming, pluripotency, and differentiation potential of bovine iPSCs by reducing ROS-induced DNA damage through increased *HIF1A* expression [9]. However, reports on how *MBD3* KD affects differentiation ability, especially PGCLC differentiation, are rare. In this study, for the first time, we identified the positive role of *MBD3* KD in the differentiation of bovine iPSCs into PGCLCs. We found that bovine iPSCs generated by MBD3 KD could produce more PGCLCs during differentiation, whereas bovine iPSCs generated without *MBD3* KD produced almost no PGCLCs during differentiation. *MBD3*-KD bovine iPSC-derived PGCLCs possess the characteristics of in vivo primordial germ cells (PGCs) in a migratory state. Transcriptomic comparison across species revealed that PGCLC-siMBD3 cells are transcriptionally closer to endogenous bovine, goat, porcine, human, and mouse PGCs than PGCLC-NC cells. The distinct PGCLC cluster (*TFAP2C+*, *PRDM1+*, *SOX15+*, and *PDPN+*) generated from iPSC-siMBD3 cultures, together with the expression of genome-stabilizing genes (*KRT8*, *RADX*, and *CRYAB*) and epigenetic modifiers (*JMJD1C* and *EZH2*) and the absence of late/meiotic markers, confirms an early PGCLC identity. These results indicate that *MBD3* knockdown during somatic cell reprogramming enhances the fidelity of the resulting iPSCs for germline differentiation.

The PGCLC induction protocol used in this study was adapted from the established two-step differentiation system originally developed for human iPSCs by Sasaki et al. (2015) [2], wherein pluripotent stem cells are first converted to incipient mesoderm-like cells (iMeLCs) via Activin A and CHIR99021 treatment, followed by exposure to BMP4, LIF, SCF, and EGF. This contrasts with the recently published protocol by Shirasawa et al. (2024) [39], which employed a fibronectin-based preinduction with concurrent BMP4, CHIR99021, and IWR1 treatment, followed by aggregate culture under sustained Wnt modulation. While both protocols successfully generate PGCLCs from bovine pluripotent stem cells, they represent distinct methodological strategies rooted in different theoretical frameworks: our approach follows the “epiblast-intermediate” paradigm, whereas Shirasawa’s approach relies on “concurrent Wnt pathway modulation” tailored to primed bESCs [39]. The convergence of both protocols on BMP4/LIF/SCF/EGF-driven germline specification underscores the central role of these cytokines in bovine PGC fate induction, while the divergence in preinduction requirements highlights the cell-line-specific plasticity of signaling responses and the value of complementary methodological approaches for the field. A limitation of the current study is the lack of direct comparison with earlier bovine PGC stages. Future work incorporating transcriptomic profiling of pre-gonadal bovine PGCs will be essential to more precisely define the developmental stage of in vitro-derived PGCLCs. *MBD3* KD increased NODAL signal transduction during the induction of PGCLCs and facilitated the expression of PRDM1 and SOX15 in bovine PGCLCs, and *MBD3* KD increased the bivalent modification of TGF-β-related genes by upregulating the expression of DPPA4 and DNMT3L in bovine iPSCs, which accounted for the increase in NODAL signaling. BMP, NODAL, and WNT signal transductions are important for PGC specification. *MBD3* knockdown accelerates and amplifies the TFAP2C-SOX15-PRDM1 cascade during bovine iPSC differentiation into PGCLCs, resulting in increased expression of TFAP2C, PRDM1, and SOX15 during differentiation and maintenance of their expression upon culture expansion. This finding suggests that *MBD3* depletion not only enhances the efficiency of PGCLC induction but also stabilizes the PGCLC transcriptional program during subsequent expansion. BMP and NODAL signaling can activate the expression of *TFAP2C* and *SOX17*, whereas the effect of WNT signaling is transient and is used to induce NODAL signaling [2,13,18]. Previous studies have shown that, when human PGCLCs are induced, WNT and NODAL signaling activates EOMES to induce *SOX17* expression, and SOX17 and TFAP2C activate the PGCLC program in a manner that is interdependent and that together promotes PRDM1 expression [14,40]. Other studies have indicated that PGCLCs require sustained BMP and NODAL expression but only require transient WNT signaling to induce NODAL signaling [13]. Our results revealed the importance of NODAL in the differentiation of iPSC-siMBD3 cells into PGCLCs. Moreover, our results indicated that, in the early stage of bovine PGCLC induction, the expression of TFAP2C is first activated, followed by the expression of SOX15, and finally, TFAP2C and SOX15 activate the expression of PRDM1. Throughout the differentiation process of PGCLCs, *TFAP2C* is dependent on BMP and WNT signaling, whereas *SOX15* is dependent on BMP and NODAL signaling. Importantly, NODAL signaling also directly increased *PRDM1* expression in PGCLC-siMBD3 cells but not in PGCLC-NC cells. *MBD3* knockdown streamlines the signaling logic of PGCLC specification: TFAP2C and SOX15 retain their dependence on BMP/WNT and BMP/NODAL signaling, respectively, whereas PRDM1 switches from multi-pathway control to NODAL-dominant signaling at the lineage-specification stage. Notably, PRDM1 exhibits transient feedback activation upon early BMP or NODAL withdrawal, suggesting a complex regulatory dynamics during PGCLC commitment. This refined signaling architecture may reduce lineage noise and promote robust PGCLC specification. Therefore, further work is needed to fully understand the function of NODAL and its specific downstream target genes.

The paradoxical coexistence of bivalent H3K4me3 and H3K27me3 domains has been identified and is generally thought to play an important role in maintaining developmental genes in a silent state primed for rapid activation during subsequent differentiation [41,42]. The total number of bivalent genes in preimplantation embryos is much lower than that in ESCs or TSCs, but the total number of bivalent genes in human PGCs is much lower than that in gonadal somatic cells and ESCs. Although trends in the number of bivalent genes during development are elusive, the dynamic changes in bivalent promoters are certainly dependent on the occupancy and removal of the H3K27me3 mark [43,44]. Mouse PGCs exhibit high levels of H3K27me3 in genes and retrotransposons enriched for developmental and differentiation functions, indicating that key developmental pathways in these germ cells are extensively silenced. However, the overall level of H3K27me3 enrichment in human PGCs is low [41,44], which may be caused by differences between species. Our results show that knocking down MBD3 during somatic cell reprogramming reduces the number of bivalent genes obtained by iPSCs, but the degree of pluripotency is greater. Therefore, whether the number of bivalent genes is related to pluripotency during embryonic development remains unknown.

There is an association between bivalent epigenetic marks and DNA methylation, and DPPA2/4 and DNMT3L serve as a bridge between H3K4me3 and H3K27me3 modifications and DNA methylation. Specifically, bivalent modification is established in part through Dppa2/4 binding to members of the COMPASS and PRC2 complexes, which deposit H3K4me3 and H3K27me3 histone modifications, respectively. In ESCs, Dppa2/4 stabilizes H3K4me3 levels and prevents de novo DNA methylation by inhibiting the binding of DNMT3L and DNA methyltransferase [45,46]. With respect to bivalent genes, Dnmt3L antagonizes the DNA methylation activity of Dnmt3a/3b and competes with Dnmt3a/3b for interaction with the PRC2 complex [29]. These two studies reached the same conclusion through different approaches, both confirming that the promoter region of bivalent genes relies on the combination of DPPA2/4 and DNMT3L to maintain a hypomethylated state. However, the specific mode of action and sequence of DPPA2/4 and DNMT3L have not been reported. Our study confirmed that a reduction in MBD3 expression increases the expression of DPPA4 and DNMT3L. DPPA4 first binds to the H3K4me3-marked promoter, stabilizes H3K4me3 levels, and prevents de novo DNA methylation; then, the reduction in MBD3 inhibits the binding of DNMT3L and HDAC1, promoting the binding of DNMT3L to the promoter of the DPPA4-bound H3K4me3 mark to establish the H3K27me3 mark and antagonize DNMT3A/B to prevent de novo DNA methylation. *MBD3* KD promotes the differentiation of bovine iPSCs into PGCLCs through the establishment of bivalent H3K4me3/H3K27me3 modifications at TGF-β-related gene promoters. This epigenetic priming mechanism, mediated by upregulated DPPA4 and DNMT3L expression, poises key developmental loci for rapid activation upon differentiation cues, thereby accelerating NODAL signaling and facilitating PGCLC commitment. Consistently, further functional validation, including *DPPA4* or *DNMT3L* knockdown and rescue experiments in MBD3-deficient cells, is required to definitively confirm their causal roles in regulating PGCLC developmental competence. Therefore, the current DPPA4/DNMT3L epigenetic regulatory model remains associative and requires further mechanistic verification in future studies.

While our transcriptomic and protein-level analyses support the PGCLC identity of the induced cells, functional validation of the capacity of these cells to contribute to gonadal development or undergo meiosis remains to be demonstrated. This limitation is shared by all current bovine PGCLC studies, as no group has yet reported complete in vitro gametogenesis from livestock PGCLCs [39]. Future studies should systematically compare the efficiency, transcriptomic fidelity, and developmental potential of PGCLCs generated through these complementary approaches, potentially leading to an optimized consensus protocol for bovine germline induction.

## 5. Conclusions

In this study, we investigated the role of MBD3 in the differentiation of bovine iPSCs into PGCLCs using a monolayer culture system. Our findings demonstrate that *MBD3* knockdown during iPSC reprogramming significantly enhances the efficiency of PGCLC induction and promotes the acquisition of migratory PGC-like characteristics. Mechanistically, *MBD3* knockdown upregulates NODAL signaling, which in turn facilitates the expression of core PGC regulators, including *PRDM1* and *SOX15*. Additionally, *MBD3* knockdown induces bivalent histone modifications at TGF-β-related gene loci and elevates the expression of pluripotency/germline-associated factors *DPPA4* and *DNMT3L*, suggesting that *MBD3*-knockdown epigenetic remodeling during reprogramming predisposes iPSCs toward a germline-competent state. Collectively, these results reveal a previously unrecognized function of MBD3 in bovine germline specification and highlight the critical impact of MBD3-dependent chromatin regulation on histone modification patterns during early germ cell development.

## Figures and Tables

**Table 1:** Expression profiles of genes associated with DNA methylation, H3K9me2, and H3K27me3 in the PGCLCs cluster in PGCLC-siMBD3 cells at day 5 of differentiation.

	PGCLCs Cluster (Total Number = 1354)	
Genes	Number of Positive Cells	Percentage of Positive Cells
DNA methylation		
*DNMT1*	1028	76.0
*DNMT3A*	303	22.3
*DNMT3B*	39	2.9
*TET1*	198	14.6
*TET2*	216	16.0
*TET3*	34	3.5
H3K9me2		
*SETDB1*	226	16.7
*KDM3A*	293	21.6
*KDM3B*	119	8.8
*JMJD1C*	1127	83.2
*KDM4A*	26	1.9
*KDM4B*	39	2.9
*KDM4C*	159	11.7
H3K27me3		
*EZH1*	24	1.8
*EZH2*	611	45.1
*KDM6A*	511	37.7
*KDM6B*	371	27.4

**Table 2:** Expression profiles of pluripotent genes, early PGCs, late PGCs, surface markers, and meiosis-related genes in PGCLC-siMBD3 cells on day 5 of differentiation.

PGCLCs Cluster (Total Number = 1354)		
Genes	Number of Positive Cells	Percentage of Positive Cells
Pluripotency		
*SALL4*	273	20.2
*KLF4*	557	41.1
*LIN28A*	75	5.5
*OCT4*	0	0
*NANOG*	10	0.74
Early PGCs		
*PRDM1*	132	9.7
*TFAP2C*	341	25.2
*SOX15*	142	10.5
*ALPL*	86	6.4
*DPPA3*	91	6.7
*CXCR4*	82	6.1
Late PGCs		
*DDX4*	1	0
*DAZL*	1	0
*PIWIL2*	0	0
Surface proteins		
*KIT*	73	5.4
*PDPN*	682	50.4
Meiosis		
*STRA8*	81	6.0
*SYCP1*	3	0.2
*SYCP2*	4	0.2
*SYCP3*	10	0.7
*STAG3*	2	0.1
*Co-expressing TFAP2C, PRDM1, SOX15, and PDPN*	23	1.7

## Data Availability

The data presented in this study are openly available through the China National Center for Bioinformation at https://ngdc.cncb.ac.cn/gsub/submit/bioproject/subPRO091309, accessed on 29 August 2026, reference number PRJCA062437.

## Supplementary Materials

The following supporting information can be downloaded at https://www.mdpi.com/article/10.3390/cells15171577/s1. Figure S1: Expression of PGC markers in PGCLCs obtained by 3D differentiation method and expression of SOX17 in PGCLCs obtained by 2D differentiation method; Figure S2: The expansion culture of bovine PGCLCs obtained by 2D method; Figure S3: Expression of PGC marker genes and proteins in EBs; Figure S4: Differences between iPSC-NC and iPSC-siMBD3 cells in the process of PGCLCs-induced differentiation; Figure S5: Comparison of bovine PGCLCs in vitro, human and mouse PGCLCs in vitro, goat and pig PGCs in vivo; Figure S6: Comparison of bovine PGCLC-NC and PGCLC-siMBD3 cells; Figure S7: Function of NODAL, BMP and WNT signals in bovine PGCLCs; Figure S8: Expression of H3K4me3 and H3K27me3 in iPSCs and during PGCLCs differentiation; Figure S9: Comparative analysis of H3K4me3, H3K27me3 and bivalent mark genes; Table S1: Primer sequences for qRT–PCR analysis; Table S2: Primary antibodies used for immunostaining; Table S3: Secondary antibodies used for immunostaining; Table S4: Primary antibodies used for western blot; Table S5: Secondary antibodies used for western blot.
