# Supplementary material for: MBD3 Knockdown Promotes the Differentiation of Bovine iPSCs into Primordial Germ Cell-like Cells Associated with Altered Bivalent Modification of TGF-β-Related Genes
Source: Cells. 2026 Aug 30;15(17):1577. doi: 10.3390/cells15171577 (PMC13564770; doi:10.3390/cells15171577)
Supplement: Supplementary file 1 [file cells-15-01577-s001.zip › cells-4415953-supplementary.pdf]

Supplementary Materials

Supplementary Materials and Methods

Induction of PGCLCs from bovine iPSCs (3D)

Bovine iPSCs were generated and cultured in LCDM system using previously published protocols[1]. The LCDM medium contained equal amounts of DMEM/F12 (Gibco) and Neurobasal (Gibco) supplemented with 0.5% N2 supplement (Gibco), 1% B27 supplement (Gibco), 1% L-glutamine (Sigma-Aldrich), 1% MEM non-essential amino acid solution (NEAA; Sigma-Aldrich), 0.1 mM β-mercaptoethanol (Sigma-Aldrich), 1% penicillin–streptomycin (PS; Gibco), 5% knockout serum replacement (KSR; Gibco), 10 ng/mL recombinant human leukemia inhibitory factor (LIF, Peprotech), 1 μM CHIR99021 (MCE), 2 μM (S)-(+)-dimethindene maleate (R&D Systems), and 2 μM minocycline hydrochloride (MCE).

Bovine iPSCs differentiate into iMeLCs and PGCLCs using previously published method[2]. For iMeLCs induction, the iPSCs were dissociated into single cells with TrypLE Select (Gibco), and 2×10^5 cells per well were plated onto of Matrigel (Millipore) coated 12-well plate in Glasgow minimum essential medium (GMEM; Gibco) supplemented with 15% KSR (Gibco), 1% NEAA (Sigma-Aldrich), 2 mM L-glutamine (Gibco), 1 mM sodium pyruvate (Gibco), 0.1 mM β-mercaptoethanol (Sigma-Aldrich), 50 ng/mL Activin A (R&D Systems), 1 μM CHIR99021 (MCE), and 10 μM ROCK inhibitor (Y-27632; MCE). After 2 days of culture, iMeLCs were digested using TrypLE Select, and then 3×10^3 cells were seeded into low-attachment 96-well plates. The cell culture medium was replaced with GMEM supplemented with 15% KSR, 0.1 mM NEAA, 2 mM L-glutamine, 1 mM sodium pyruvate, 0.1 mM β-mercaptoethanol, 200 ng/mL bone morphogenetic protein 4 (BMP4; MCE), 100 ng/mL stem cell factor (SCF; Peprotech), 20 ng/mL LIF (Millipore), 50 ng/mL epidermal growth factor (EGF; R&D Systems), and 10 μM Y-27632 (MCE) for PGCLCs induction. The medium was changed daily. The culture was continued for 6-8 days.

Expansion culture of bovine PGCLCs

On the 6th day of PGCLC differentiation, cells were washed with DPBS, and Tryple Select was used to dissociate the culture into single cells. Then cells were seeded into 12-well plates coated with Matrigel or gelatin at a passage

ratio of 1:4. The culture medium contains Dulbecco's modified eagle medium (DMEM; Gibco) supplemented with 15% KSR (Gibco), 2.5% fetal bovine serum (FBS; Bovoge), 1% NEAA (Sigma-Aldrich), 2 mM L-glutamine (Gibco), 0.1 mM β-mercaptoethanol (Sigma-Aldrich), 1% PS (Gibco), 200 ng/mL SCF (PeproTech), 20 ng/mL fibroblast growth factor 2 (FGF2; PeproTech), 10 μM Forskolin (MCE), and 10 μM Y-27632 (MCE). The medium was changed every day. Passage was performed every 4 days.

Alkaline phosphatase (AP) staining

The AP activity of bovine PGCLCs was analyzed using the BCIP/NBT Alkaline Phosphatase Chromogenic Kit (Beyotime Biotechnology), following the manufacturer's instructions. In short, cell culture medium was removed, and the cells were washed once with DPBS. 4% paraformaldehyde was added and fixed for 10 min, and the cells were washed three times with DPBS. The staining solution was prepared and added to the cells, and stained for 15 min at room temperature in the dark. Then the cells were washed with DPBS three times to completely remove the staining solution and observed the staining results under a microscope.

Embryoid body (EB) formation

Bovine iPSCs were subjected to enzymatic digestion to obtain single cells. These cells were subsequently cultured in improved minimum essential medium (IMDM; Gibco) medium supplemented with 15% FBS at 38.5 °C under 5% CO_2. On days 4-6, EBs gradual formed and were transferred onto gelatin-coated 4-well plates. The medium was replaced every two days with fresh DMEM (Gibco) containing 15% FBS. Subsequently, on days 26-30, the cells were utilized for immunofluorescence staining and real-time PCR analyses.

Supplementary Figures S1 to S9

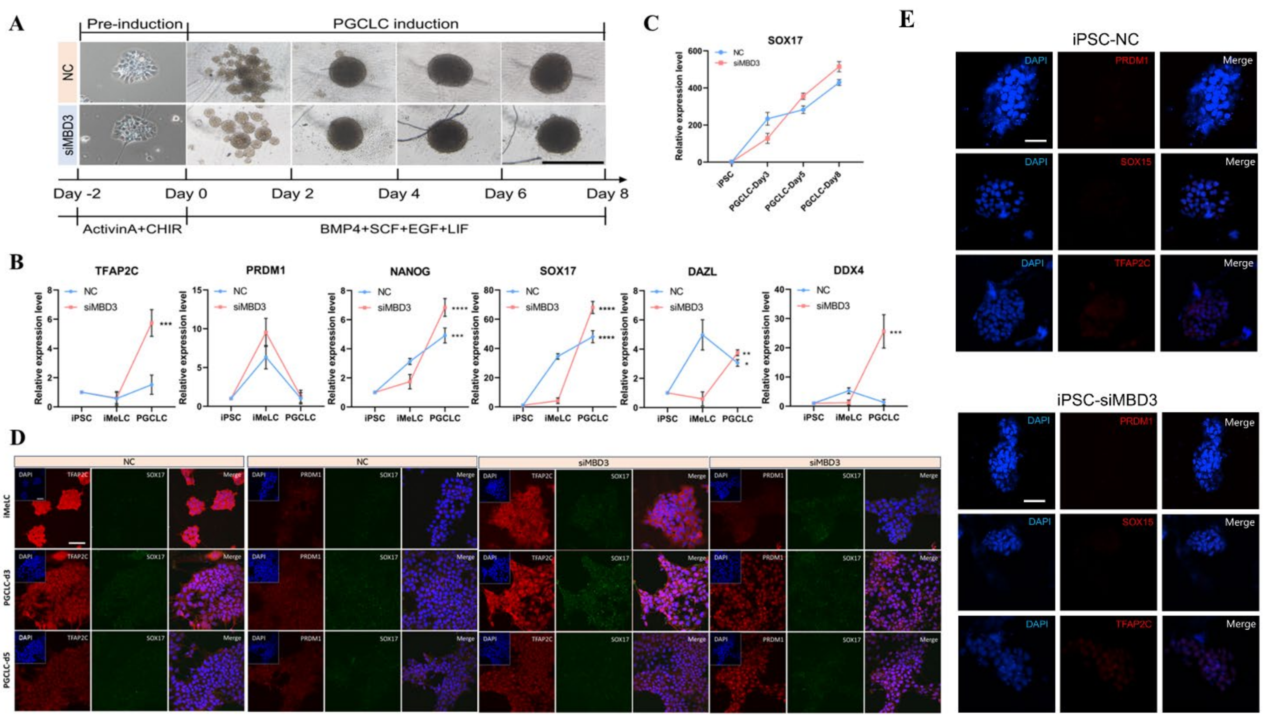

Figure S1 Expression of PGC markers in PGCLCs obtained by 3D differentiation method and expression of SOX17 in PGCLCs obtained by 2D differentiation method. (**A**) The diagram of PGCLCs induction through 3D method. Scale bars, 100 μm. (**B**) Expression of pluripotency and PGC marker genes during 3D differentiation of bovine PGCLCs. (**C**) Expression of *SOX17* gene during 2D differentiation of bovine PGCLCs. (**D**) Co-immunofluorescence staining of PGC markers during 2D differentiation of bovine PGCLCs. (**E**) Immunofluorescence staining of PGCLC markers (*TFAP2C*, *PRDM1*, and *SOX15*) in iPSC-NC and iPSC-siMBD3. Nuclei were stained with DAPI. Scale bars, 50 μm.

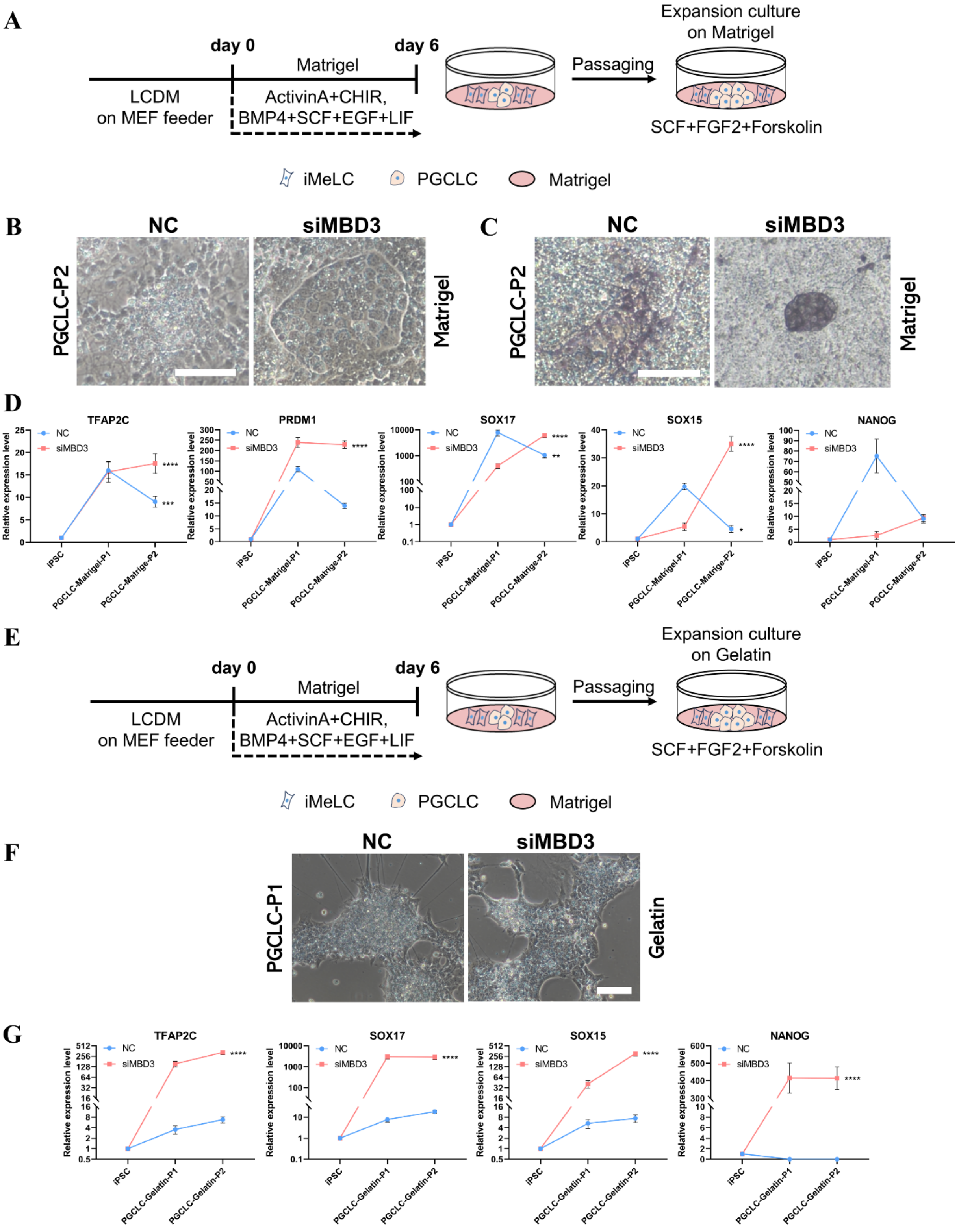

Figure S2 The expansion culture of bovine PGCLCs obtained by 2D method. (**A**) Schematic diagram of the expansion culture of bovine PGCLCs obtained by the 2D method on Matrigel. (**B**) Morphology of bovine PGCLCs obtained by 2D method in subculture and expansion culture on Matrigel. Scale bar: 100 μm. (**C**) AP staining of bovine PGCLCs obtained

by 2D method inthe expansion culture on Matrigel. Scale bar: 100 μm. (**D**) Expression of pluripotency and PGCs marker genes (*TFAP2C*, *PRDM1*, *SOX15*, *SOX17*, *NANOG*) inthe expansion culture on Matrigel. (**E**) Schematic diagram of the expansion culture of bovine PGCLCs obtained by the 2D method on gelatin. (**F**) Morphology of bovine PGCLCs obtained by 2D method in the expansion culture on gelatin. Scale bar: 100 μm. (**G**) Expression of pluripotency and PGCs marker genes (*TFAP2C*, *SOX15*, *SOX17*, *NANOG*) inthe expansion culture on gelatin.

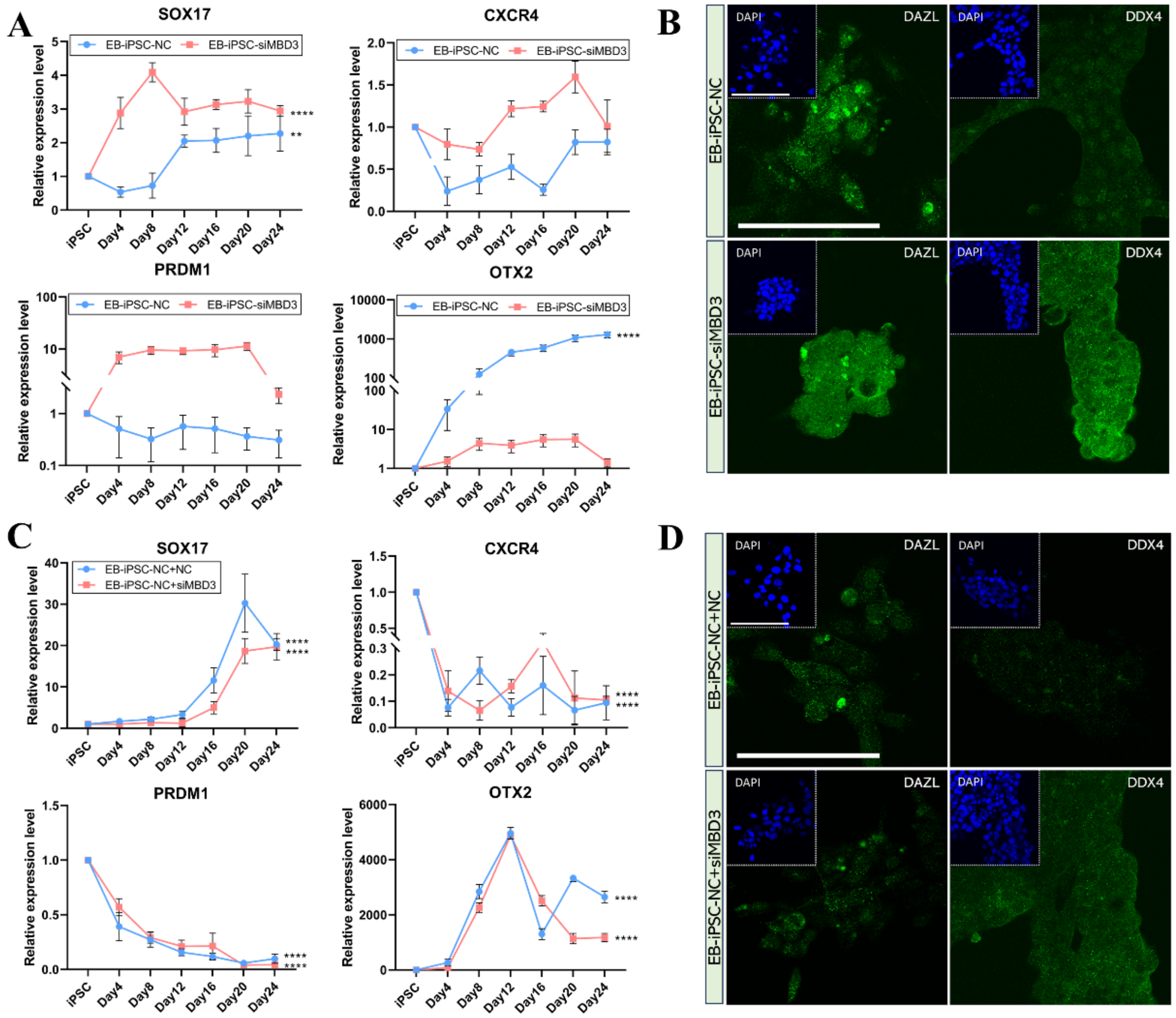

Figure S3 Expression of PGC marker genes and proteins in EBs. (**A**) Expression of *SOX17* and related genes during EBs differentiation of bovine iPSC-NC cells. (**B**) Immunofluorescence analysis of DAZL and DDX4 in EBs. Nuclei were stained with DAPI. Scale bars, 100 μm. (**C**) Expression of *SOX17* and related genes during EBs differentiation of MBD3 knockdown iPSC-NC cells. (**D**) Immunofluorescence analysis of DAZL and DDX4 in EBs (MBD3 knockdown iPSC-NC). Nuclei were stained with DAPI. Scale bars, 100 μm.

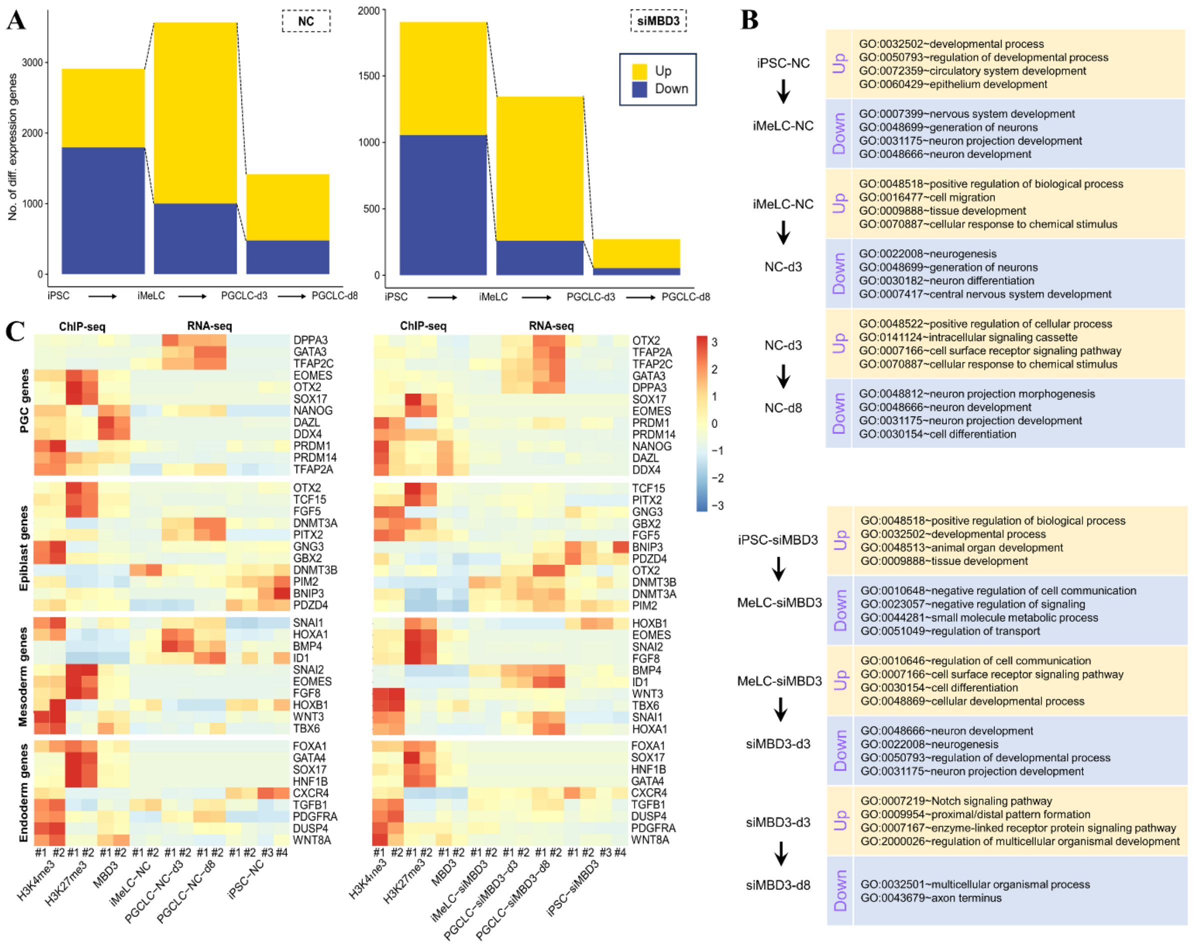

Figure S4 Differences between iPSC-NC and iPSC-siMBD3 cells in the process of PGCLCs-induced differentiation. (**A**) Number of up- or down-regulated genes of different stages of PGCLCs differentiation (fold change > 2, FDR < 0.01). (**B**) GO analyses of the genes shown in (A) during PGCLC specification. (**C**) Heat map of normalized expression of selected genes related to ectoderm, mesoderm and endoderm in bovine iPSCs, iMeLCs and PGCLCs.

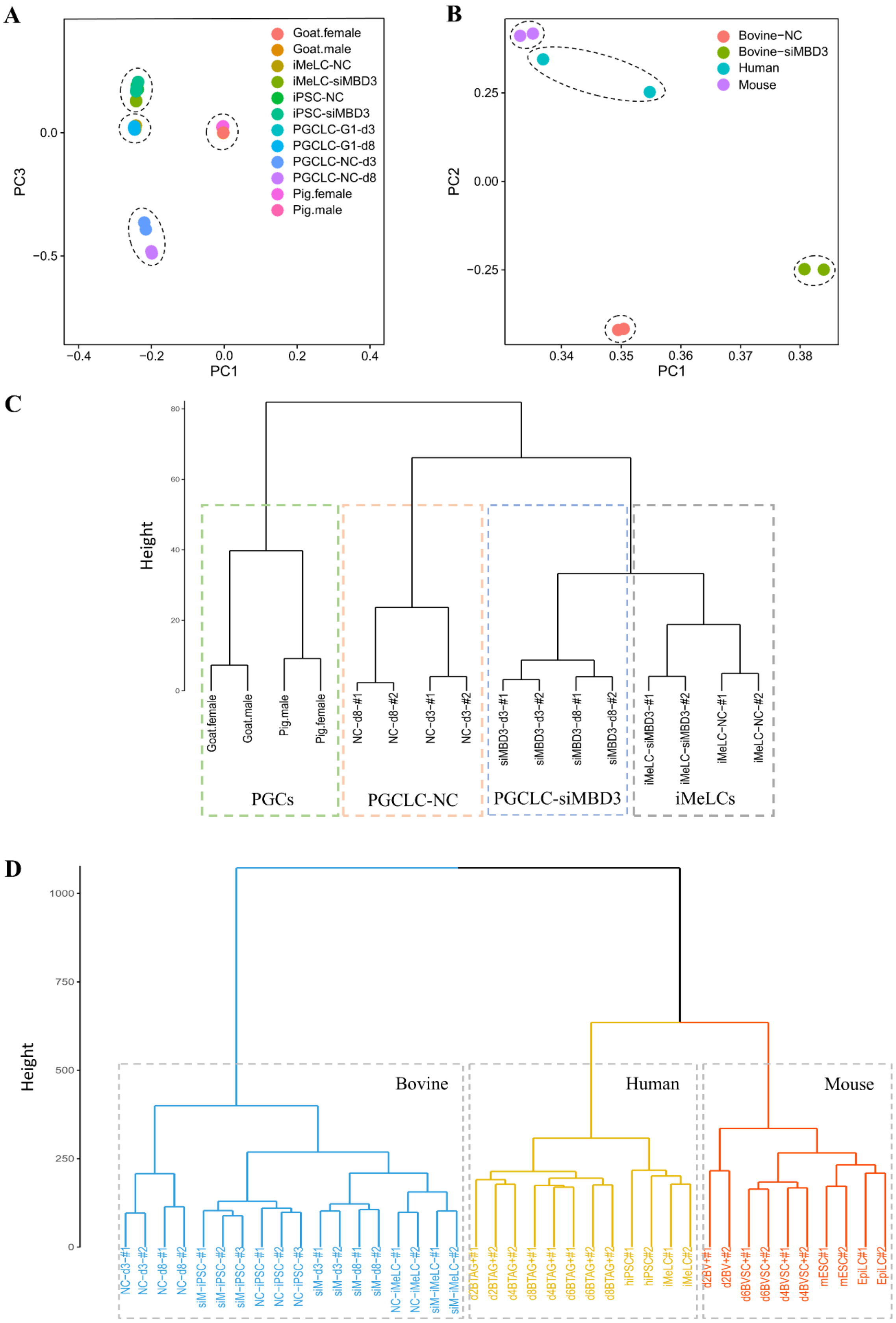
A
Goat.female
Goat.male
iMeLC-NC
iMeLC-siMBD3
iPSC-NC
iPSC-siMBD3
PGCLC-G1-d3
PGCLC-G1-d8
PGCLC-NC-d3
PGCLC-NC-d8
Pig.female
Pig.male
PC3
PC1
B
Bovine−NC
Bovine−siMBD3
Human
Mouse
PC2
PC1
C
Height
Goat.female
Goat.male
Pig.male
Pig.female
NC−d8−#1
NC−d8−#2
NC−d3−#1
NC−d3−#2
siMBD3−d3−#1
siMBD3−d3−#2
siMBD3−d8−#1
siMBD3−d8−#2
iMeLC−siMBD3−#1
iMeLC−siMBD3−#2
iMeLC−NC−#1
iMeLC−NC−#2
PGCs
PGCLC-NC
PGCLC-siMBD3
iMeLCs
D
Height
Bovine
Human
Mouse
NC−d3−#1
NC−d3−#2
NC−d8−#1
NC−d8−#2
siM−iPSC−#1
siM−iPSC−#2
siM−iPSC−#3
NC−iPSC−#1
NC−iPSC−#2
NC−iPSC−#3
siM−d3−#1
siM−d3−#2
siM−d8−#1
siM−d8−#2
NC−iMeLC−#1
NC−iMeLC−#2
siM−iMeLC−#1
siM−iMeLC−#2
d2BTAG+#1
d2BTAG+#2
d4BTAG+#2
d8BTAG+#1
d4BTAG+#1
d6BTAG+#1
d6BTAG+#2
d8BTAG+#2
hiPSC#1
hiPSC#2
iMeLC#1
iMeLC#2
d2BV+#1
d2BV+#2
d6BVSC+#1
d6BVSC+#2
d4BVSC+#1
d4BVSC+#2
mESC#1
mESC#2
EpiLC#1
EpiLC#2

Figure S5 Comparison of bovine PGCLCs in vitro, human and mouse PGCLCs in vitro, goat and pig PGCs in vivo. (**A**) PCA of bovine PGCLCs, goat PGCs, and pig PGCs. (**B**) PCA of bovine PGCLCs, human PGCLCs, and mouse PGCLCs. (**C**) UHC of bovine PGCLCs, goat PGCs, and pig PGCs. (**D**) UHC of bovine PGCLCs, human PGCLCs, and mouse PGCLCs.

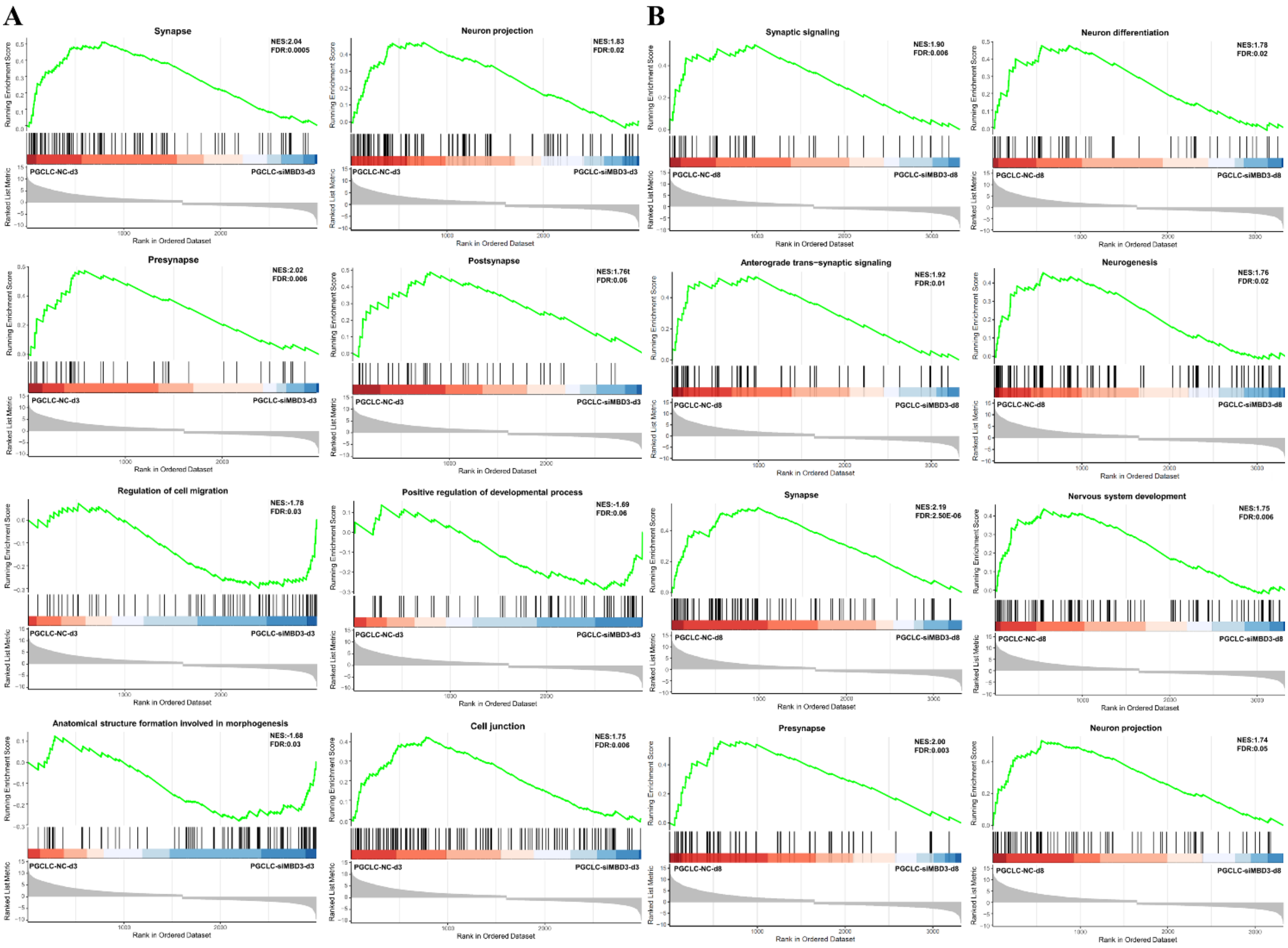

Figure S6 Comparison of bovine PGCLC-NC and PGCLC-siMBD3 cells. (**A**) GSEA of PGCLC-NC and PGCLC-siMBD3 cells on the third day of induction. (**B**) GSEA of PGCLC-NC and PGCLC-siMBD3 cells on the eighth day of induction.

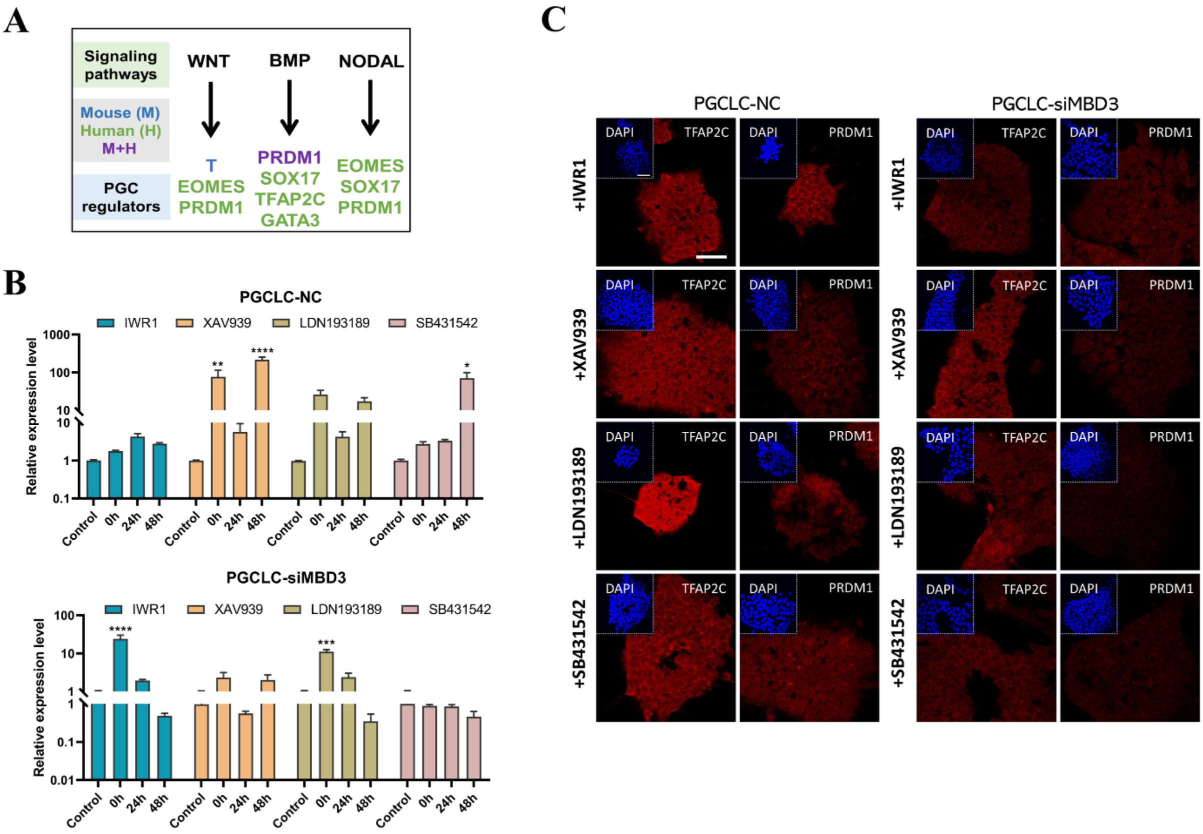

Figure S7 Function of NODAL, BMP and WNT signals in bovine PGCLCs. (**A**) Downstream factors regulated by NODAL, BMP and WNT signaling in published literature. Blue: validated at the mouse cell level[3], green: validated at the human cell level[4-6], purple: validated at both mouse and human cell levels[3]. (**B**) Changes of *SOX17* gene expression after adding signaling pathway inhibitors. (**C**) Immunofluorescence staining of PGCs markers after adding inhibitors. Nuclei were stained with DAPI. Scale bars, 100 μm.

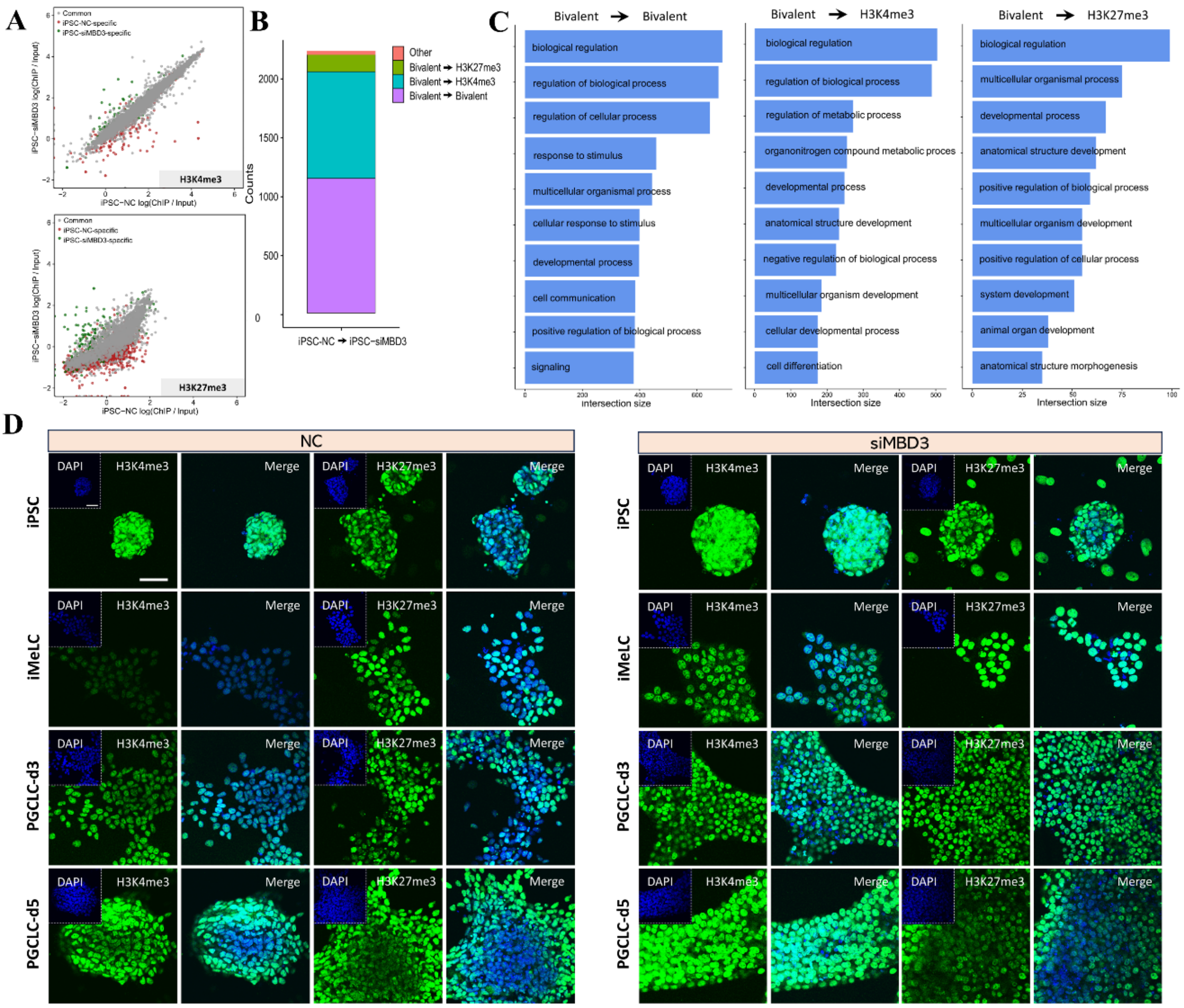

Figure S8 Expression of H3K4me3 and H3K27me3 in iPSCs and during PGCLCs differentiation. (**A**) Scatter plot showing the relative expression of H3K4me3 and H3K27me3 only modified genes in bovine iPSC-NC and iPSC-siMBD3 cells. Highlight genes modified only with H3K4me3 or H3K27me3. Genes that remain unchanged are colored in gray. (**B**) Comparison of bivalent gene status in bovine iPSC-NC and iPSC-siMBD3 cells. The number of genes whose chromatin state changes from bivalent to the indicated state is shown. (**C**) GO term analyses based on the genes identified in (B). Top 10 significant terms are shown. (**D**) Immunofluorescence staining of H3K4me3 and H3K27me3 at different stages. Nuclei were stained with DAPI. Scale bars, 100 μm.

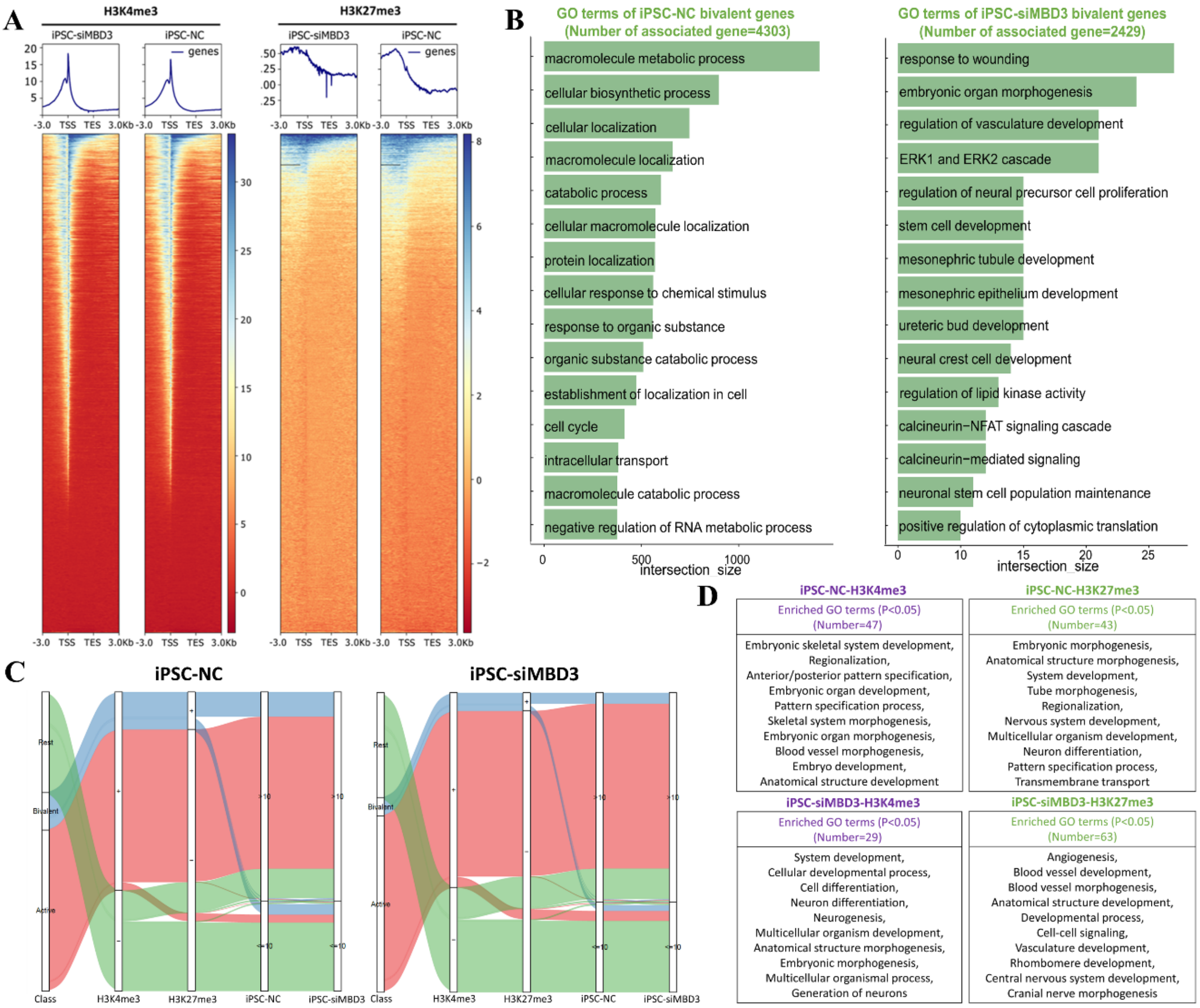

Figure S9 Comparative analysis of H3K4me3, H3K27me3 and bivalent mark genes. (**A**) Genes bound by H3K4me3 (left) or H3K27me3 (right) in iPSC-NC and iPSC-siMBD3 cells. Shown is a 3 kb window centered on the peak. (**B**) GO term analysis was performed based on genes with bivalent modifications (H3K4me3 and H3K27me3). (**C**) Alluvial plot showing the overall dynamics of divalently labeled regions in iPSCs. (**D**) GO term analysis was performed on genes with H3K4me3 or H3K27me3 modification based on enrichment analysis.

Supplementary Tables S1 to S5

Table S1 Primer sequences for qRT–PCR analysis.

Gene	Forward primer	Reverse primer
b-GAPDH	GGGTCATCATCTCTGCACCT	GGTCATAAGTCCCTCCACGA
b-SOX17	AAGATGCTGGGCAAGTCG	CGGTACTTGTAGTTGGGATGG

b-TFAP2C	CGACATGGCACACCAGAT	GGAAATAGGACCTTTGCGAATAAC
b-PRDM1	ATGGAGGACGCGGATATGAC	TGTTGGCGTTCTTGGGAACT
b-NANOG	TTCCCTCCTCCATGGATCTG	ATTTGCTGGAGACTGAGGTA
b-CXCR4	AGGAAGCTGCTGGCTGAAAA	CCTTGCGCTTCTGATAGCCT
b-OTX2	GGCTGAGTCTGACCACTTCG	CTCGGGTGAATGTCGTCCTC
m-Acrosin	CACGTGTGGACCTCATTGAC	GTAGTCCCAGGTGGCTGTGT
m-Lhcgr	CGCTTTCCAAGGGATGAATA	CTGGAGGGCAGAGTTTTCAG
m-Stella	CTTTGTTGTCGGTGCTGAAA	TCCCGTTCAAACTCATTTCC
m-Sox17	GGAGGGTCACCACTGCTTTA	AGATGTCTGGAGGTGCTGCT
m-Nanog	GGGCTATCTGGTGAACGCAT	CCCGAAGTTATGGAGCGGAG
h-SOX17	TTCGTGTGCAAGCCTGAGAT	TAATATACCGCGGAGCTGGC
h-TFAP2C	ATTAAGAGGATGCTGGGCTCTG	CACTGTACTGCACACTCACCTT
h-BLIMP1	AAACCAAAGCATCACGTTGACA	GGATGGATGGTGAGAGAAGCAA
h-NANOS3	TGGCAAGGGAAGAGCTGAAATC	TTATTGAGGGCTGACTGGATGC
h-NANOG	ACAACTGGCCGAAGAATAGCA	GGTTCCCAGTCGGGTTCAC

Table S2 Primary antibodies used for immunostaining.

Protein	Species	Dilution	Catalog #	Vendor
TFAP2C	Rabbit	1:150	bs-6694R	Bioss
SOX17	Mouse	1:100	sc-130295	Santa Cruz Biotechnology
PRDM1	Rabbit	1:150	bs-6466R	Bioss
SOX15	Rabbit	1:150	16725-1-AP	Proteintech
DAZL	Rabbit	1:150	abs121836	Absin
DDX4	Rabbit	1:150	ab13840	Abcam
H3K4me3	Rabbit	1:150	9751S	Cell Signaling Technology
H3K27me3	Rabbit	1:150	9733S	Cell Signaling Technology

Table S3 Secondary antibodies used for immunostaining.

Protein	Species	Dilution	Catalog #	Vendor
FITC anti-mouse	Goat	1:500	AP130F	Millipore

Alexa Fluor 568 anti-rabbit	Goat	1:500	A11036	Life technology
Alexa Fluor 488 anti-rabbit	Donkey	1:500	A21206	Life technology

Table S4 Primary antibodies used for western blot.

Protein	Species	Dilution	Catalog #	Vendor
GAPDH	Rabbit	1:5000	10494-1-AP	Proteintech
DNMT3A	Rabbit	1:1000	D23G1	Cell Signaling Technology
DNMT3B	Rabbit	1:800	bs-0301R	Bioss
HDAC1	Mouse	1:1000	A18304	ABclonal
MBD3	Rabbit	1:1000	99169S	Cell Signaling Technology
β-CATENIN	Rabbit	1:1000	9562S	Cell Signaling Technology
pβ-CATENIN	Rabbit	1:500	28853-1-AP	Proteintech
pSMAD2	Rabbit	1:1000	AP1342	ABclonal
pSMAD3	Rabbit	1:1000	9520S	Cell Signaling Technology
SMAD2/3	Rabbit	1:1000	8685T	Cell Signaling Technology
pSMAD1	Rabbit	1:2000	80257-1-RR	Proteintech
pSMAD5	Rabbit	1:1000	AP1411	ABlonal
SMAD1/5	Rabbit	1:200	bs-2973R	Bioss
DNMT3L	Rabbit	1:1000	A13591	ABclonal
DPPA2	Rabbit	1:500	12689-1-AP	Proteintech
DPPA4	Rabbit	1:500	17045-1-AP	Proteintech

Table S5 Secondary antibodies used for western blot.

Protein	Dilution	Catalog #	Vendor
anti-rabbit IgG HRP	1:4000	W402B	Promega
anti-mouse IgG HRP	1:4000	W401B	Promega
